# Meeting abstracts from the first European Emergency Medical Services congress (EMS2016): Copenhagen, Denmark. 30 May - 1 June 2016

**DOI:** 10.1186/s13049-017-0358-0

**Published:** 2017-02-22

**Authors:** Alexander White, Han Xian Ng, Wai Yee Ng, Eileen Kai Xin Ng, Stephanie Fook-Chong, Phek Hui Jade Kua, Marcus Eng Hock Ong, Alvilda Thougaard Steensberg, Lars Bredevang Andersen, Mette Mølby Eriksen, Ole Mazur Hendriksen, Thomas Thougaard, Andreas Claesson, J. Lennartsson, L. Svensson, M. Ringh, J. Hollenberg, P. Nordberg, M. Rosenqvist, T. Djarv, S. Österberg, D. Fredman, Y. Ban, Anne-Sophie Löwe, Jacob Nielsen, Martin Zimling, Jakob Schmidt, Freddy Lippert, Annette K. Ersbøll, Thea Palsgaard Møller, Mikkel D. Jørgensen, Freddy Lippert, Annika Hamilton, Jacob Steinmetz, Mads Wissenberg, Christian Torp-Pedersen, Freddy Lippert, Lars Hove, Nicolai Lohse, Bodil Thorsager, Hanne Bonde, Maria Birkvad Rasmussen, Doris Østergaard, Camilla Schade Hansen, Mikkel Brabrand, Annmarie Lassen, Camilla Schade Hansen, Mikkel Brabrand, Annmarie Lassen, Cilia Kjer, Mathias Holgersen, Sandra Viggers, Claus Kjær Pedersen, Morten Thingemann Bøtker, Ingunn Skogstad Riddervold, Christian Juhl Terkelsen, Dag S. Nystøyl, S. Hunskaar, E. Zakariassen, David Fredman, Leif Svensson, Yifang Ban, Martin Jonsson, Jacob Hollenberg, Per Nordberg, Mattias Ringh, Mårten Rosenqvist, Margareta Lundén, Andreas Claesson, David Fredman, Martin Jonsson, Jan Haas, Leif Svensson, Yifang Ban, Andreas Claesson, Filipe Ribeiro, Mark Newton, Paulo Freitas, Dario Rocha, Emilio Leal, Nuno Santos, Tania Cortez, Stephanie Allmark, Janet Marsden, Gitte Linderoth, Freddy Lippert, Thea Palsgaard Møller, Doris Østergaard, Grethe Thomas, Anne Møller Nielsen, Gertrud Øllgaard, Hideo Inaba, Akira Yamashita, Testuo Maeda, Håkon Kvåle Bakke, Tine Steinvik, Johan Angell, Torben Wisborg, Håkon Kvåle Bakke, Tine Steinvik, Håkon Ruud, Torben Wisborg, Ingunn Anda Haug, Tonje S. Birkenes, Helge Myklebust, Jo Kramer-Johansen, Kamilia S. Funder, Lars S. Rasmussen, Rasmus Hesselfeldt, Volkert Siersma, Nicolai Lohse, Asger Sonne, Sandra Wulffeld, Jacob Steinmetz, amilia S. Funde, Lars S. Rasmussen, Nicolai Lohse, Rasmus Hesselfeldt, Volkert Siersma, Frants Pedersen, Ole M. Hendriksen, Freddy K. Lippert, Jacob Steinmetz, Kim Sol-A, Sand Do Shin, Kyungwon Lee, Eui Jung Lee, Young Sun Ro, Ki Jeong Hong, Yu Jin Kim, Joo Jeong, Park Jeong Ho, Lars Grassmé Binderup, Søren Mikkelsen, Caroline Schaffalitzky de Muckadell, Hans Morten Lossius, Palle Toft, Annmarie Touborg Lassen, Lee Thompson, Michael Hill, Maren Ranhoff Hov, Thomas Lindner, Eirik Franer, Andreas Monstad, Christian G. Lund, Martin Betzer, Rasmus Malmberg Lyngby, Milla Jousi, Jouni Nurmi, Nanna Kruse, Charlotte Barfod, Nikolaj Raaber, Morten T. Bøtker, Sophie-Charlott Seidenfaden, Ingunn S. Riddervold, Paul Simpson, Liz Thyer, Ben van Nugteren, Sophie-Charlott Seidenfaden, Ingunn S. Riddervold, Hans Kirkegaard, Niels Juul, Morten T. Bøtker, T. Zwisler, Camilla Rønnov, Hanne B. Mieritz, Søren Mikkelsen, Gitte Jørgensen, Susanne Ångerman-Haasmaa, Sami Länkimäki, Jouni Nurmi, Søren Mikkelsen, Hans Morten Lossius, Palle Toft, Annmarie Touborg Lassen, Søren Viereck, Thea Palsgaard Møller, Josephine Philip Rothman, Fredrik Folke, Freddy K. Lippert, Teodora Filipescu, Alasdair Gray, Teresa A Williams, Kwok M. Ho, Hideo Tohira, Daniel Fatovich, Deon Brink, Paul Bailey, Gavin D. Perkins, Judith Finn, Thea Palsgaard Møller, Søren Viereck, Fredrik Folke, Freddy Lippert, Thea Palsgaard Møller, Annette Kjær Ersbøll, Thora Majlund Kjærulff, Doris Østergaard, Janne Shurmann Tolstrup, Jon Trærup Andersen, Jerry Overton, Lars S. Rasmussen, Fredrik Folke, Freddy Lippert, Theo Walther Jensen, Thea Palsgaard Møller, Søren Viereck, Jens Roland, Fredrik Folke, Jens Flensted Lassen, Doris Østergaard, Freddy Lippert, Tuukka Puolakka, Sami Länkimäki, Jyrki Puolakka, Juhana Hallikainen, Kirsi Rantanen, Markku Kuisma

**Affiliations:** 1 Health Services Research, Division of Research, Singapore General Hospital, Singapore, Singapore; 2 London School of Medicine, London, UK; 3 Unit for Pre-hospital Emergency Care - Clinical Support Team, Singapore General Hospital, Singapore, Singapore; 4 Centre for Quantitative Medicine, Duke-NUS Graduate Medical School, Singapore, Singapore; 5 Department of Emergency Medicine, KK Women’s and Children’s Hospital, Singapore, Singapore; 6 Department of Emergency Medicine, Singapore General Hospital, Singapore, Singapore; 7 Health Services and Systems Research, Duke-NUS Graduate Medical School, Singapore, Singapore; 8 Prehospital Center, Region Zeeland, Denmark; 9 Slagelse Hospital, Slagelse, Denmark; 10 Karolinska Institutet, Department of Medicine, Solna, Center for Resuscitation Science, Stockholm, Sweden; 11 The Royal institute of technology (KTH), School of Architecture and the Built Environment, Department of Urban Planning and Environment, Division of geoinformatics, Stockholm, Sweden; 12 Karolinska Institutet, Department of Clinical Science, Danderyd University Hospital, Stockholm, Sweden; 13 Emergency Medical Services Copenhagen, University of Copenhagen, Copenhagen, Denmark; 14 Unit of Quality and Patient Safety, Copenhagen, Denmark; 15 Falck Emergency, Copenhagen, Denmark; 16 National Institute of Public Health, University of Southern Denmark, Copenhagen, Denmark; 17 Emergency Medical Services Copenhagen, University of Copenhagen, Copenhagen, Denmark; 18 Department of Anaesthesiology and Intensive Care, Hvidovre Hospital, University of Copenhagen, Copenhagen, Denmark; 19 Emergency Medical Services Copenhagen, University of Copenhagen, Copenhagen, Denmark; 20 Department of Anaesthesia, Centre of Head and Orthopaedics, Rigshospitalet, University of Copenhagen, Copenhagen, Denmark; 21 The Institute of Health, Science and Technology, Aalborg University, Aalborg, Denmark; 22 Copenhagen Academy for Medical Education and Simulation, Copenhagen, Denmark; 23 Department of Emergency Medicine, Odense University Hospital, Odense, Denmark; 24 Department of Emergency Medicine, Hospital of South West Jutland, Esbjerg, Denmark; 25 Department of Emergency Medicine, Odense University Hospital, Odense, Denmark; 26 Department of Emergency Medicine, Hospital of South West Jutland, Esbjerg, Denmark; 27 SATS, Students’ Society for Anesthesiology & Traumatology, Copenhagen, Denmark; 28 Copenhagen Academy for Medical Education and Simulation, Herlev, Denmark; 29 Department of Cardiology, Aarhus University Hospital, Skejby, Denmark; 30 Prehospital Emergency Medical Services, Aalborg, Central Denmark Region Denmark; 31 Department of Research, Norwegian Air Ambulance Foundation, Drøbak, Norway; 32 National Centre for Emergency Primary Health Care, Uni Research Health, Bergen, Norway; 33 Research Group for General Practice, Department of Global Public Health and Primary Care, University of Bergen, Bergen, Norway; 34 Karolinska Institutet, Department of Medicine, Solna & Center for resuscitation Science Södersjukhuset, Stockholm, Sweden; 35 KTH Royal Institute of Technology, Department of Urban Planning & Environment, Division of Geoinformatics, Stockholm, Sweden; 36 Karolinska Institutet, Department of clinical sciences, Danderyds sjukhus, Stockholm, Sweden; 37 SOS alarm AB, Dispatch Center, Stockholm, Sweden; 38 Karolinska Institutet, Department of Medicine, Solna & Center for Resuscitation Science Södersjukhuset, Stockholm, Sweden; 39 KTH Royal Institute of Technology, Department of Urban Planning & Environment, Division of Geoinformatics, Stockholm, Sweden; 40 Serviço Regional de Protecção Civil e Bombeiros dos Açores, Lisbon, Portugal; 41 North West Ambulance Service, Manchester, UK; 42 Hospital Dr. Fernando da Fonseca, Grupo Português de Triagem, Lisbon, Portugal; 43 Manchester Metropolitan University, Manchester, UK; 44 Emergency Medical Services Copenhagen, University of Copenhagen, Copenhagen, Denmark; 45 Copenhagen Academy for Medical Education and Simulation, Herlev, Denmark; 46 TrygFonden, Virum, Denmark; 47 Department of Anaesthesia, Centre of Head and Orthopaedics, Copenhagen University Hospital, Rigshospitalet, Copenhagen, Denmark; 48 NIRAS, Allerød, Denmark; 49 Department of Emergency Medical Science, Kanazawa University Graduate School of Medicine, Kanazawa, Japan; 50 Department of Cardiology, Noto General Hospital, Nanao, Japan; 51 Mo i Rana Hospital, Helgeland Hospital Trust, Mo i Rana, Norway; 52 Anaesthesia and Critical Care Research Group, Faculty of Health Sciences, University of Tromsø, Tromsø, Norway; 53 Lawyers Leiros & Olsen AS, Tromsø, Norway; 54 Faculty of Law, University of Tromsø, Tromsø, Norway; 55 Hammerfest Hospital, Department of Anaesthesiology and Intensive Care, Finnmark Health Trust, Hammerfest, Norway; 56 Norwegian National Advisory Unit on Trauma, Division of Emergencies and Critical Care, Oslo University Hospital, Oslo, Norway; 57 Mo i Rana Hospital, Helgeland Hospital Trust, Mo i Rana, Norway; 58 Anaesthesia and Critical Care Research Group, Faculty of Health Sciences, University of Tromsø, Tromsø, Norway; 59 Clinic of Emergency Medical Services, University Hospital of Northern Norway Harstad, UNN Hospital Trust, Harstad, Norway; 60 Hammerfest Hospital, Department of Anaesthesiology and Intensive Care, Finnmark Health Trust, Hammerfest, Norway; 61 Norwegian National Advisory Unit on Trauma, Division of Emergencies and Critical Care, Oslo University Hospital, Oslo, Norway; 62 Laerdal Medical, Stavanger, Norway; 63 Norwegian National Advisory Unit on Prehospital Emergency Medicine (NAKOS), Oslo, Norway; 64 Department of Anaesthesia, Centre of Head and Orthopaedics, Copenhagen University Hospital Rigshospitalet, Copenhagen, Denmark; 65 Department of Neuroanaesthesiology, Neuroscience Centre, Copenhagen University Hospital, Rigshospitalet, Copenhagen, Denmark; 66 The Research Unit for General Practice and Section of General Practice, Department of Public Health, University of Copenhagen, Copenhagen, Denmark; 67 Department of Anaesthesia, Centre of Head and Orthopaedics, Copenhagen University Hospital Rigshospitalet, Copenhagen, Denmark; 68 Department of Neuroanaesthesiology, Neuroscience Centre, Copenhagen University Hospital, Rigshospitalet, Copenhagen, Denmark; 69 The Research Unit for General Practice and Section of General Practice, Department of Public Health, University of Copenhagen, Copenhagen, Denmark; 70 Department of Cardiology, The Heart Centre, Copenhagen University Hospital, Rigshospitalet, Copenhagen, Denmark; 71 Prehospital Centre, Copenhagen, Region Zealand Denmark; 72 Emergency Medical Services Copenhagen, University of Copenhagen, Copenhagen, Denmark; 73 Laboratory of Emergency Medical Service, Bio-Medical Research Institute, Seoul National University Hospital, Seoul, South Korea; 74 Mobile Emergency Care Unit, Department of Anaesthesiology and Intensive Care Medecine V, Odense University Hospital, Odense, Denmark; 75 Philosophy, Department for the Study of Culture, University of Southern Denmark, Odense, Denmark; 76 Prehospital Critical Care, Stavanger University, Stavanger, Norway; 77 Emergency Medicine Research Unit Odense University Hospital, Odense, Denmark; 78 North East Ambulance Service, Newcastle upon Tyne, UK; 79 Northumbria University, Newcastle upon Tyne, UK; 80 The Norwegian Air Ambulance Foundation, Grålum, Norway; 81 Østfold Hospital, Grålum, Norway; 82 Prehospital Centre, Region Zealand, Denmark; 83 Emergency Medical Services Copenhagen, Copenhagen, Denmark; 84 Department of Emergency Medicine, Helsinki University Hospital and University of Helsinki, Helsinki, Finland; 85 Department of Aneasthesia and Intensive Care, Hillerød Hospital, Hillerød, Denmark; 86 Emergency Medical Services Copenhagen, University of Copenhagen, Copenhagen, Denmark; 87 Department of Research and Development, Prehospital Emergency Medical Services, Aarhus, Central Denmark Region Denmark; 88 Western Sydney University, Sydney, Australia; 89 University of Johannesburg, Johannesburg, South Africa; 90 Department of Research and Development, Prehospital Emergency Medical Services, Aarhus, Central Denmark Region Denmark; 91 Research Centre for Emergency Medicine, Aarhus University Hospital, Aarhus, Denmark; 92 Department of Neuro Anaesthesiology, Aarhus University Hospital, Aarhus, Denmark; 93 Department of Anesthesiology and Intensive Care, Odense University Hospital, Odense, Denmark; 94 Dispatch Central, Odense, Region of Southern Denmark Denmark; 95 University of Southern Denmark, Odense, Denmark; 96 Mobile Emergency Care Unit Region of Southern Denmark, Odense, Denmark; 97 Department of Emergency Medicine, Helsinki University Hospital and University of Helsinki, Helsinki, Finland; 98 Mobile Emergency Care Unit, Department of Anaesthesiology and Intensive Care Medecine, Odense University Hospital, Odense, Denmark; 99 Prehospital Critical Care, Stavanger University, Stavanger, Norway; 100 Emergency Medicine Research Unit, Odense University Hospital, Odense, Denmark; 101 Emergency Medical Services, University of Copenhagen, Copenhagen, Denmark; 102 Center for Perioperative Optimization, Department of Surgery, Herlev Hospital, University of Copenhagen, Copenhagen, Denmark; 103 University of Edinburgh, Edinburgh, Scotland; 104 Emergency Medicine NHS Lothian, Edinburgh, Scotland; 105 Prehospital Resuscitation and Emergency Care Research Unit, Curtin University, Perth, Australia; 106 St John Ambulance-Western Australia, Perth, Australia; 107 Royal Perth Hospital, Perth, Australia; 108 Intensive Care Unit, Royal Perth Hospital, Perth, Australia; 109 School of Population Health, The University of Western Australia, Perth, Australia; 110 Emergency Medicine, Royal Perth Hospital, Perth, Australia; 111 Centre for Clinical Research in Emergency Medicine, Harry Perkins Institute of Medical Research, Nedlands, Australia; 112 Critical Care Medicine, Warwick Medical School, University of Warwick, Coventry, UK; 113 Heart of England NHS Foundation Trust, Birmingham, UK; 114 Emergency Medical Services, Copenhagen and University of Copenhagen, Copenhagen, Denmark; 115 Emergency Medical Services, Copenhagen and University of Copenhagen, Copenhagen, Denmark; 116 National Institute of Public Health, University of Southern Denmark, Copenhagen, Denmark; 117 Copenhagen Academy for Medical Education and Simulation, Herlev Hospital, University of Copenhagen, Copenhagen, Denmark; 118 Laboratory of Clinical Pharmacology, Copenhagen University Hospital Rigshospitalet, Copenhagen, Denmark; 119 International Academies of Emergency Dispatch, Salt Lake City, Utah USA; 120 Centre of Head and Orthopaedics, Copenhagen University Hospital Rigshospitalet, Copenhagen, Denmark; 121 Emergency Medical Services Copenhagen, University of Copenhagen, Copenhagen, Denmark; 122 ERC CPR-AED-group Denmark - The Danish Emergency Management Agency, Birkerød, Denmark; 123 Department of Cardiology, Aarhus University Hospital, Aarhus, Denmark; 124 Danish Resuscitation Council, Copenhagen, Denmark; 125 Copenhagen Academy for Medical Education and Simulation, Herlev Hospital, University of Copenhagen, Copenhagen, Denmark; 126 Helsinki University Hospital, Helsinki, Finland

## A1 Measuring the effectiveness of a novel CPRcard feedback device during simulated chest compressions by non-healthcare workers

### Alexander White^1^*, Han Xian Ng^2^, Wai Yee Ng^1^, Eileen Kai Xin Ng^3^, Stephanie Fook-Chong^1,4^, Phek Hui Jade Kua^5^, Marcus Eng Hock Ong^6,7^

#### ^1^Health Services Research, Division of Research, Singapore General Hospital, Singapore, Singapore; ^2^London School of Medicine, London, UK; ^3^Unit for Pre-hospital Emergency Care - Clinical Support Team, Singapore General Hospital, Singapore, Singapore; ^4^Centre for Quantitative Medicine, Duke-NUS Graduate Medical School, Singapore, Singapore; ^5^Department of Emergency Medicine, KK Women’s and Children’s Hospital Singapore, Singapore, Singapore; ^6^Department of Emergency Medicine, Singapore General Hospital, Singapore, Singapore; ^7^Health Services and Systems Research, Duke-NUS Graduate Medical School, Singapore, Singapore

##### **Correspondence:** Alexander White (alexander.elgin.white@sgh.com.sg)

**Background**

We aimed to see if a novel credit card size cardiopulmonary resuscitation (CPR) feedback device helped to improve the quality of chest compressions by lay participants compared to compressions done without feedback.

**Materials and methods**

This study had non-healthcare workers aged 25 – 70 years old randomized into either a real-time feedback group that got the CPRcard, which provided real-time feedback for both chest compression rate and depth, or the no feedback group. Participants in the control group (no feedback) either used a blinded CPRcard or performed compressions without a CPRcard.

**Results**

Participants in the CPRcard group achieved a better median compression rate (CPRcard: 117 vs control: 122, p-value = 0.001) and higher proportion of compressions within the adequate range of 100 to 120 per minute (CPR Card: 83% vs control: 47%, p-value < 0.001). CPRcard group had a higher percentage of adequate compressions (CPRcard: 88% vs. no card: 46.8%, p-value = 0.037; CPRcard: 73% vs blinded card: 43%, p-value = 0.003). The participants in the CPRcard group more often performed better quality CPR, defined as simultaneously meeting targets for both compression rate of 100 to 120 and depth of at least 5cm (CPRcard: 36% vs control: 4%, p-value = 0.022).

**Conclusions**

Use of the CPRcard by non-healthcare workers improved the quality of CPR chest compressions.

## A2 Bystander capability to activate speaker function for continuous telephone CPR in case of suspected cardiac arrest

### Alvilda Thougaard Steensberg^1^*, Lars Bredevang Andersen^1,2^, Mette Mølby Eriksen^1^, Ole Mazur Hendriksen^1^, Thomas Thougaard^1,2^

#### ^1^Prehospital Center, Region Zeeland, Slagelse, Denmark; ^2^Slagelse Hospital, Slagelse, Denmark

##### **Correspondence:** Alvilda Thougaard Steensberg (alvilda.steensberg@gmail.com)

**Background**

Medical emergency dispatchers should provide telephone-CPR in all cases of suspected cardiac arrest, unless a trained provider is already delivering CPR. If the bystanders phone has a speaker facility the ERC guidelines recommendation is to switch it on as this will facilitate continuous dialogue with the emergency medical dispatcher including (if required) CPR instructions. This is standard procedure in our organization, but until now, we have only found evidence about bystander capability to activate speaker in test settings.

**Materials and methods**

In a 30-day period a systematic prospective registration of the bystander capability to activate the speaker was done. Included are all calls with suspected cardiac arrest. From these calls, the cases when it was obviously out of scope to ask were excluded and the reasons were registered. The bystanders were asked: “Does your phone have a speaker function?” If yes: “Can you turn it on?” The emergency medical dispatcher afterwards registered if it succeeded.

**Results**

Out of approximately 5600 calls, 96 were coded as suspected cardiac arrest. In 45 cases it was out of scope to ask. 66.7% of the asked cases, where the phone had a speaker function, were able to turn the speaker on.

**Conclusions**

66.7% success rate of switching to speaker function is better than expected, but there is room for improvement. Cardiac arrest can be a stressful situation. That is why telephone CPR with speaker function needs more attention and could be a focus point in future CPR courses.

## A3 Potential of unmanned aerial vehicles (UAV) to save lives in simulated out-of-hospital-cardiac-arrest - the Drone trial

### Andreas Claesson^1^*, J. Lennartsson^2^, L. Svensson^1^, M. Ringh^1^, J. Hollenberg^1^, P. Nordberg^1^, M. Rosenqvist^3^, T. Djarv^1^, S. Österberg^1^, D. Fredman^1^, Y. Ban^2^

#### ^1^Karolinska Institutet, Department of Medicine, Solna, Center for Resuscitation Science, Stockholm, Sweden; ^2^The Royal Institute of Technology (KTH), School of Architecture and the Built Environment, Department of Urban Planning and Environment, Division of Geoinformatics, Stockholm, Sweden; ^3^Karolinska Institutet, Department of Clinical Science, Danderyd University Hospital, Stockholm, Sweden

##### **Correspondence:** Andreas Claesson (andreas.claesson@telia.com)

**Background**

Early cardiopulmonary resuscitation (CPR) and use of an automated external defibrillator (AED) can increase 30-day survival from 10% up to 70%. An unmanned aerial vehicle (UAV) might have a role in transporting an AED to the site of an OHCA. The aim of this experimental study was to describe the potential benefit of an UAV system for delivery of an AED in a rural environment.

**Materials and methods**

Optimal placement and response times for AED equipped UAV were calculated using GIS-models based on two weighting alternatives. UAV delivery testflights were performed using three different techniques.

**Results**

All OHCA cases with a cardiac aetiology n = 7923 in Stockholm county 2006-2013 were analyzed. Ten optimal locations with a 10 km radius in the greater Stockholm area were identified for implementation of UAV systems. With a simulated 50/50 weighting n = 7905 cases were found primarily in the city centre. The UAV arrived before EMS in 32% of cases with a mean timesaving of 1.5 min. With a simulated 80/20 weighting including n = 134 OHCA cases in primarily remote areas, the UAV arrived before EMS in 93% of cases with a mean timesaving of 19 min. Delivery of the AED in testflights n = 14 was successful in favourable conditions within sight primarily by latch-released technique or by landing the UAV on flat ground.

**Conclusions**

By using GIS models optimal placement of UAV systems can be calculated. These locations might in the future significantly reduce time to defibrillation and serve as a complement to EMS services.

## A4 Prehospital patient safety incidents – a description based on a national mandatory reporting system

### Anne-Sophie Löwe^1^*, Jacob Nielsen^2^, Martin Zimling^1^, Jakob Schmidt^3^, Freddy Lippert^1^

#### ^1^Emergency Medical Services Copenhagen, University of Copenhagen, Copenhagen, Denmark; ^2^Unit of Quality and Patient Safety, Copenhagen, Denmark; ^3^Falck Emergency, Copenhagen, Denmark

##### **Correspondence:** Anne-Sophie Löwe (anne-sophie.kyndlo.loewe.02@regionh.dk)

**Background**

Registration and analysis of patient safety incidents (PSIs) is recommended as a method to improve patient safety in the prehospital setting – a method used in-hospital. Research concerning prehospital patient safety is sparse. In Denmark, a mandatory reporting system has been implemented for the Emergency Medical Services (EMS) since 2011 to register prehospital PSIs. This study aims to describe two years of reported prehospital PSIs in EMS Copenhagen based on PSI reports from the Danish mandatory reporting system.

**Materials and methods**

The Capital Region of Denmark serves a population of 1,7million and approx. 180,000 ambulance tasks are being performed yearly. PSI reports regarding cases related to the ambulance service in the Capital Region from the years 2013-2014 were included and categorized into 14 main categories based on the primary issue. The profession of the reporter and the location of the incidents were noted.

**Results**

In total, 264 PSI were reported (1 : 1400 cases). The majority were concerning: “Equipment” (29.2%), “Organization” (14.8%), “Prehospital treatment” (13.6%), “Medication” (11.7%), and “Interhospital transportation” (8.3%). The PSIs were mainly reported by the ambulance personnel (EMTs and paramedics; 58.0%) or nurses from hospitals (28.8%). 84.5% of cases concerned ambulances and 11% included the physician staffed mobile critical care unit.

**Conclusions**

The implementation of a prehospital reporting system to PSIs was successful, and 264 events have been reported in 2 years in EMS Copenhagen. The most frequent category reported are safety issues concerning equipment. Most of the PSIs are reported by the ambulance personnel themselves.

## A5 Geographical clustering of service goal fulfillment for emergency ambulances in the Capital Region of Denmark

### Annette K Ersbøll^1^*, Thea Palsgaard Møller^2^, Mikkel D. Jørgensen^2^, Freddy Lippert^2^

#### ^1^University of Southern Denmark, National Institute of Public Health, Copenhagen, Denmark; ^2^Emergency Medical Services Copenhagen, University of Copenhagen, Copenhagen, Denmark

##### **Correspondence:** Annette K Ersbøll (ake@niph.dk)

**Background**

Ambulance response time (RT) is a common quality indicator in Emergency Medical Services (EMS). Service goals have been developed to increase the EMS quality, including RT target values. Target values were: 13 min and 25 min for 90% of the ambulances dispatched with highest priority 1 and priority 2, respectively. We aimed at examining and identifying areas with significantly higher proportion of calls with RT above the recommended target values.

**Materials and methods**

A total of 146,256 calls were included in the analysis from the Capital Region of Denmark with a catchment area of 1.7 mill individuals. Data were electronically collected and included geographical location of pick-up, priority response level, and RT. Small-area estimation methods were used to estimate smooth maps of the geographical distribution of the proportion of calls with RT above the target values. Spatial clustering was performed using spatial scan statistics to identify areas with significantly higher proportions of calls with RT above the target values.

**Results**

The median RT was 6:05 min and above the target value in 5.6% of priority 1 ambulances. For priority 2 ambulances, the median RT was 13:25 min and 12.4% were above the target value. Areas with significantly higher proportions of calls with RT above the target values were identified for both response priority levels with some overlap between the clusters.

**Conclusions**

Using geographical methods areas with a significantly higher proportion of long RT were identified and can be used to allocate resources to improve RT in certain areas.

## A6 Association between prehospital physician involvement and survival after out-of-hospital cardiac arrest: a Danish nationwide observational study

### Annika Hamilton^1^*, Jacob Steinmetz^2,3^, Mads Wissenberg^2^, Christian Torp-Pedersen^4^, Freddy Lippert^2^, Lars Hove^1,2^, Nicolai Lohse^1,3^*

#### ^1^Department of Anaesthesiology and Intensive Care, Hvidovre Hospital, University of Copenhagen, Copenhagen, Denmark; ^2^Emergency Medical Services Copenhagen, University of Copenhagen, Copenhagen, Denmark; ^3^Department of Anaesthesia, Centre of Head and Orthopaedics, Rigshospitalet, University of Copenhagen, Copenhagen, Denmark; ^4^The Institute of Health, Science and Technology, Aalborg University, Aalborg, Denmark

##### **Correspondence:** Annika Hamilton (niclohse@gmail.com)

**Background**

Sudden out-of-hospital cardiac arrest (OHCA) is an important public health problem. While several indicators are known to improve survival, the impact of physician-delivered advanced cardiac life support for OHCA is unclear. We aimed to assess the association between prehospital physician involvement and 30-day survival.

**Materials and methods**

Observational study including persons registered with first-time OHCA of any cause in the Danish Cardiac Arrest Registry during 2005-2012. The association between 30-day survival and involvement of a physician at any time before arrival at the hospital was assessed in a multivariable propensity score-matched logistic regression model. Secondary outcomes were 1-year survival and return of spontaneous circulation (ROSC) before arrival at the hospital.

**Results**

21,165 persons with OHCA during 2005-2012 were included. The proportion of OHCAs with physician involvement increased from 57.1% in 2005 to 67.9% in 2012 (test for trend, p < 0.001). During the same time period, 30-day survival increased from 5.8% in 2005 to 11.5% in 2012 (p < 0.001). Overall, 10.8% of OHCA patients with physician involvement and 8.1% of OHCA patients without physician involvement before arrival at hospital were alive after 30 days, crude Odds Ratio (OR) = 1.37(95% CI = 1.24-1.51), adjusted OR = 1.18 (95% CI = 1.04-1.34). Physician involvement was also positively associated with ROSC upon arrival at the hospital, OR = 1.09 (95% CI = 1.00-1.19); and with 1-year survival, OR = 1.13 (95% CI = 0.99-1.29).

**Conclusions**

In this large population-based observational study, we found prehospital physician involvement after OHCA associated with better 30-day survival. This association was also found for ROSC on arrival, but with less certainty for 1-year survival.

## A7 The development and delivery of a course for coordinating teams in the Emergency department

### Bodil Thorsager*, Hanne Bonde, Maria Birkvad Rasmussen, Doris Østergaard

#### Copenhagen Academy for Medical Education and Simulation, Copenhagen, Denmark

##### **Correspondence:** Bodil Thorsager (bodil.thorsager@dadlnet.dk)

**Background**

The aim of the educational endeavour was to develop and deliver a course to improve communication, coordination and workflow in the coordinating teams of physicians and nurses in an Emergency Department.

**Materials and methods**

A needs analysis consisting of observations, workshops and discussions with management and staff members was conducted. The workshop participants mapped the workflow with related responsibilities and tasks. Challenges and suggestions for solutions were identified and discussed. Important challenges included issues related to shared decision-making and leadership, particularly when the flow of emergency patients was high. A checklist was developed to structure the communication about the patients’ conditions and staff members’ availability. A simulation based training course for the coordinating teams were developed. A screen similar to what staff use in their daily work was developed and used during the simulations to make these as similar to real life as possible. A pre – and post questionnaire to evaluate their self-assessment of competence and an evaluation form were developed (5 point Likert-scale)

**Results**

Seven sessions for 14 teams were conducted. Sixteen coordinating nurses and fourteen physicians were trained. The participants evaluated the team joint situation awareness, decision-making and leadership higher after the course. The staff evaluated the simulations as relevant and the ability to transfer learning to practice to 4.7 and 4.3, respectively.

**Conclusions**

The needs assessment provided valuable input to the content of the course. The coordinating team members evaluated the simulations as relevant and useful for the practice.

## A8 Prognosis of poisoned patients with specific ECG changes in the emergency department - a cohort study

### Camilla Schade Hansen^1^*, Mikkel Brabrand^1,2^, Annmarie Lassen^1^

#### ^1^Department of Emergency Medicine, Odense University Hospital, Odense, Denmark; ^2^Department of Emergency Medicine, Hospital of South West Jutland, Esbjerg, Denmark.

##### **Correspondence:** Camilla Schade Hansen (camilla.schade.hansen@rsyd.dk)

**Background**

Poisoning has previously been associated with specific ECG changes, however the clinical impact of these changes remains unknown. The aim of this study is to investigate if corrected QT (QTc) prolongation, QRS widening, tachycardia, and bradycardia are associated with increased mortality within 30 days after arrival to the emergency department (ED).

**Materials and methods**

A register based observational prospective cohort study, including all adult (≥18 years) first time admissions to the ED of Odense University Hospital from 1 October 2013 to 30 September 2014 with suspected poisoning. We calculated the proportion of death, including a 95% CI based on Poisson distribution, within 30 days for poisoned patients with and without an ECG change.

**Results**

597 patients arrived with suspected poisoning (52.1% men, mean age 44 SD 19), and 425/597 (71.2%) had an ECG recorded. An ECG change, defined as prolonged QTc (≥450 ms men, ≥460 ms women; Framingham formula), QRS widening (≥120 ms), tachycardia (≥100 beats/min), or bradycardia (≤50 beats/min) occurred in 125/425 (29.4%). Overall 7/425 (1.6%; 95% CI 0.7-3.4) patients died within 30 days after arrival to the ED. Among patients with an ECG change 30-days mortality was 5/125 (4%; 95% CI, 1.3-9.3) compared to 2/300 (0.7%; 95% CI 0.08-2.4) in patients without ECG changes. 31/425 (7.3%) had QTc prolongation, 2/31 (6.3%; CI 95% 0.8-23.3) died within 30 days.

**Conclusions**

We found no significant difference in 30-days mortality comparing poisoned patients with ECG changes to poisoned patients without ECG changes, however the risk of type 2 error is considered high.

## A9 A cross sectional study linking specific ECG changes to specific groups of poisonings in the emergency department

### Camilla Schade Hansen^1^*, Mikkel Brabrand^1,2^, Annmarie Lassen^1^

#### ^1^Department of Emergency Medicine, Odense University Hospital, Odense, Denmark; ^2^Department of Emergency Medicine, Hospital of South West Jutland, Esbjerg, Denmark

##### **Correspondence:** Camilla Schade Hansen **(**camilla.schade.hansen@rsyd.dk)

**Background**

Corrected QT (QTc) prolongation, QRS widening, tachycardia, and bradycardia have been described in poisonings, but prevalence in relation to specific groups of poisonings is unknown. We aimed at describing the prevalence of ECG abnormalities in specific poisonings in patients arriving to an emergency department (ED).

**Materials and methods**

A descriptive register based cross sectional study including all first time admissions among adult patients, arriving at the ED of Odense University Hospital with a diagnose of poisoning (ICD-10 code T36*-T65*), from 3 December 2012 to 30 December 2014. We divided poisonings into 5 main groups: (1) Analgesics and drugs of abuse, (2) cardiovascular, (3) psychotropic, (4) chemical and biological substances, non-medical, and (5) others.

**Results**

427 (47.8% male, mean age 42 SD 18) patients were included. 368/427 (86.2%) had an ECG recorded. QTc prolongation (Framingham formula, cutoff 460 ms women, 450 ms men) was most prevalent in group 5 (7/48; 14.6%), followed by group 4 (2/32; 6.3%), 1 (10/171; 5.8%), and 3 (3/114; 2.6%). Extreme QTc prolongation (≥500 ms) occurred in 5/368 (1.4%) of patients, distributed among group 1, 3 and 5. 11/368 (3.0%) had QRS ≥120 ms, with 4/48 (8.3%) observed in group 5. Tachycardia (≥100 beats/min) was frequent in all groups, ranging from 4/32 (12.4%) in group 4 to 26/114 (22.8%) in group 3, while bradycardia (≤50 beats/min) was infrequently observed.

**Conclusions**

Patients poisoned by miscellaneous substances had the highest prevalence of QTc prolongation while tachycardia was most common in the groups of psychotropic and analgesics including drugs of abuse.

## A10 The use of medical simulation in introducing Ebola protection procedures to health care professionals

### Cilia Kjer^1^*, Mathias Holgersen^1,2^, Sandra Viggers^1,2^

#### ^1^SATS, Students’ Society for Anesthesiology & Traumatology, Copenhagen, Denmark; ^2^Copenhagen Academy for Medical Education and Simulation, Herlev, Denmark

##### **Correspondence:** Cilia Kjer (ckwk2@hotmail.com)

**Background**

Ebola is a single-stranded RNA virus that causes haemorrhagic fever. The 2014 outbreak in West Africa resulted in >28,000 cases. The mortality rate is 29%-67% depending on the country of treatment. The importance of global preparedness was emphasized in Texas where healthcare professionals (HCP) became infected with Ebola due to insufficient use of personal protective equipment (PPE). The donning and doffing (D&D)-procedures for PPE are extensive and time consuming. This may increase the risk of HCP not adhering to guidelines if not properly trained. This abstract presents a way to use medical simulation in introducing HCP to the use of Ebola-PPE.

**Materials and methods**

We developed a medical simulation in Ebola-PPE for use at an emergency medicine course. Precourse the participants were asked to review a guideline on D&D procedures. The scenario included a simulated flight to Sierra Leone. A video demonstration of Ebola-PPE and general information about Ebola was shown during the flight.

**Results**

38 out of 60 participants evaluated the simulation. A total of 92% of the participants totally or partially agreed that the simulation have increased their knowledge. 95% totally or partially agreed that the setting of the simulation added to the realism of the scenario.

**Conclusions**

This simulation scenario is an easy and inexpensive way to educate people in Ebola-PPE and the scenario can be made in a simple classroom setting. To ensure correct learning outcome a facilitator familiar with D&D-procedures should assess the participants to ensure correct learning.

## A11 Diagnoses and mortality in EMS-callers suffering chest pain

### Claus Kjær Pedersen^1^*, Morten Thingemann Bøtker^2^, Ingunn Skogstad Riddervold^2^, Christian Juhl Terkelsen^1^

#### ^1^Department of Cardiology, Aarhus University Hospital, Skejby, Denmark; ^2^Prehospital Emergency Medical Services, Aalborg, Central Denmark Region, Denmark

##### **Correspondence:** Claus Kjær Pedersen (clapes@rm.dk)

**Background**

Chest pain might indicate life-threatening conditions, e.g. acute coronary syndrome (ACS) and acute myocardial infarction (AMI), but also a range of other low-risk conditions. In Denmark, ambulance dispatch is criteria-based. The aim of this study was to investigate the proportion of patients assigned the dispatch criteria “Chest pain – suspected heart disease” subsequently diagnosed with ACS or AMI and the associated mortality.

**Materials and methods**

Population-based follow-up study of patients calling 112 in the Central Denmark Region from October 1 2011 to December 31 2014. Diagnoses according to the 10th version of the International Classification of Disease (ICD-10) were retrieved from the Danish National Patient Registry. Vital status from the Danish Civil Registration System was retrieved using a censor date of November 26 2015. Long-term mortality was compared using Cox proportional hazards regression.

**Results**

Of the total 75,696 112-calls, 8555 patients suffered from chest pain. ACS was confirmed in 11% (n = 949), including AMI in 8% (n = 700). 30-day mortality was 1.7% (95% CI 1.4 – 2.0) in patients not diagnosed with ACS and 2.2% (95% CI 1.5 – 3.4) in patients diagnosed with ACS. Long-term mortality was higher in patients diagnosed with ACS with a HR of 1.24 (95% CI 1.04 – 1.5) compared to patients not diagnosed with ACS.

**Conclusions**

In patients calling 112 due to chest pain, only 11% is diagnosed with ACS and 8% with AMI. Mortality in these patients is slightly higher when a diagnosis of ACS is confirmed.

## A12 Transport, treatment and level of care when helicopter evacuation is not accessible; a retrospective descriptive study

### Dag S Nystøyl ^1,2^*, S. Hunskaar^2,3^, E. Zakariassen^1,2,3^

#### ^1^Department of Research, Norwegian Air Ambulance Foundation, Drøbak, Norway; ^2^National Centre for Emergency Primary Health Care, Uni Research Health, Bergen, Norway; ^3^Research Group for General Practice, Department of Global Public Health and Primary Care, University of Bergen, Bergen, Norway

##### **Correspondence:** Dag S Nystøyl **(**dag.nystoyl@uni.no)

**Background**

Air ambulances in Norway do not achieve full regularity, mainly due to weather conditions. There is lack of knowledge about transport, treatment and level of care when the air ambulance is not able to fly. The aim was to describe alternative transport and patient care.

**Materials and methods**

Data based on all rejected and aborted helicopter missions in the county of Sogn & Fjordane in Norway from 2010-2013 was obtained. Excluded were all missions cancelled due to no medical need. We obtained data from the records of the Emergency Medical Communication Centre and prehospital services. Descriptive data analyses was performed.

**Results**

We included 183 missions with 191 patients. Median age of the patients was 58 years (25%-75% percentiles: 30 – 71) and 61% was male. Of the patients 68% were located outside hospital and all was transported with ground ambulance. GP on call was alarmed in 89% and responded with a call out in 50%. A physician followed 24% of the patients during transport. Of the patients 90% was transported to a hospital. No treatment was given in 26%, while 16% was given oxygen.

**Conclusions**

In contrast to the request of air ambulance majority of the patients was treated and transported to hospital by ground ambulance without a physician involved during transport. In 42% of the patient no treatment or oxygen only was given.

## A13 Expanding the first link in the chain of survival - dispatchers referral of callers to AED locations

### David Fredman^1^*, Leif Svensson^1^, Yifang Ban^2^, Martin Jonsson^1^, Jacob Hollenberg^1^, Per Nordberg^1^, Mattias Ringh^1^, Mårten Rosenqvist^3^, Margareta Lundén^4^, Andreas Claesson^1^.

#### ^1^Karolinska Institutet, Department of Medicine, Solna & Center for resuscitation Science Södersjukhuset, Stockholm, Sweden; ^2^KTH Royal Institute of Technology, Department of Urban Planning & Environment, Division of Geoinformatics, Stockholm, Sweden; ^3^Karolinska Institutet, Department of clinical sciences, Danderyds sjukhus, Stockholm, Sweden; ^4^SOS alarm AB, Dispatch center, Stockholm, Sweden

##### **Correspondence:** David Fredman (david.a.fredman@ki.se)

**Background**

The dispatch center constitutes the first link in the chain of survival in cases of out-of-hospital cardiac arrest (OHCA). Dispatchers play an important role and should according to current guidelines provide callers with instructions in telephone-assisted cardiopulmonary resuscitation (T-CPR) and the locations of automated external defibrillators (AED). The aim of this study was to investigate if dispatchers refer callers to nearby AED in suspected OHCA when provided with a tool to identify AED locations during emergency calls.

**Materials and methods**

An application with real-time information on AED locations were provided to four dispatch centers in Sweden. Retrospectively, a geographic information system was used to identify cases of suspected OHCA located ≤100 meters from an AED and audio recordings of these calls were assessed to evaluate the rate of AED referral by dispatchers.

**Results**

3009 calls concerning suspected OHCA were handled at the four dispatch centers during seven consecutive months in 2014 and in 6.6% (200/3009) an AED was ≤100 meters of the suspected OHCA. In 24% (47/200) of these cases the AED was accessible and the caller was not alone on scene and could be referred to the AED without jeopardizing T-CPR. In 4.3% (2/47) the dispatcher referred the caller to the nearby AED.

**Conclusions**

Limited AED accessibility and the fact that the caller was alone on scene made AED referral by dispatchers rare. For such a system to be effective must not only the numbers of AED in society improve but also the access hours of these AED.

## A14 Are AED located where OHCA occur? - a mismatch in AED installation in areas with similar OHCA incidence in Stockholm Sweden

### David Fredman^1^*, Martin Jonsson^1^, Jan Haas^2^, Leif Svensson^1^, Yifang Ban^2^, Andreas Claesson^1^

#### ^1^Karolinska Institutet, Department of Medicine, Solna & Center for resuscitation Science Södersjukhuset, Stockholm Sweden; ^2^KTH Royal Institute of Technology, Department of Urban Planning & Environment, Division of Geoinformatics, Stockholm, Sweden

##### **Correspondence:** David Fredman **(**david.a.fredman@ki.se)

**Background**

Early defibrillation in out-of-hospital cardiac arrest (OHCA) increases survival. Optimal locations for automated external defibrillators (AED) are debated and the number of AED in Stockholm is increasing. Yet, annual OHCA incidence in public locations is relatively unchanged. We hypothesize that AED distribution follows the historic incident locations of public OHCA in Stockholm and aim to visualize this spatial relation and to calculate the distance between OHCA and AED.

**Materials and methods**

Geographic locations of n = 1,828 public available AED as of Dec. 31st 2013 were obtained from the Swedish AED registry and merged with coordinates of n = 804 public OHCA during 40 months in Stockholm. In areas defined by the standardized pan-European digital mapping tool, Urban Atlas (UA), OHCA and AED proportions were visualized and median distances were calculated.

**Results**

In the two areas with the highest public OHCA incidence, low-density urban fabric area (29.7%) and industrial/commercial area (28.4%) the AED distribution was 9.6% and 59.0% respectively. The median distance from OHCA to AED in these two areas were 461.9 meter and 141.6 meter respectively.

**Conclusions**

AED distribution does not follow public OHCA incidence in Stockholm County; there is a mismatch in AED distribution in areas with similar OHCA incidence and also a three-times-greater median distance. Standardized digital mapping tools like UA can be used to perform similar calculations and comparisons in or between other countries or regions. This could shine a light on low AED use by visualizing areas of mismatch with high OHCA incidence and low AED numbers.

## A15 Clinical Telephone Triage and Advice in the Azorean archipelago: establishing the clinical utility and safety of the Manchester Triage System

### Filipe Ribeiro^1^*, Mark Newton^2^, Paulo Freitas^3^, Dario Rocha^1^, Emilio Leal^1^, Nuno Santos^1^, Tania Cortez^1^, Stephanie Allmark^2^, Janet Marsden^4^

#### ^1^Serviço Regional de Protecção Civil e Bombeiros dos Açores, Lisbon, Portugal; ^2^North West Ambulance Service, Manchester, UK; ^3^Hospital Dr. Fernando da Fonseca, Grupo Português de Triagem, Lisbon, Portugal; ^4^Manchester Metropolitan University, Manchester, UK

##### **Correspondence:** Filipe Ribeiro **(**Filipemribeiro@gmail.com)

**Background**

The Azorean archipelago consists of nine islands spread across 600 kilometers and whilst call volumes are manageable, the need to manage resources effectively is crucial. The primary objective of the study was to implement a new telephone triage system, evaluate its safety, and the resulting impact on pre-hospital emergency response in the Azores.

**Materials and methods**

Over 18 months, patients accessing care via 112 were triaged trough the Manchester Triage System Telephone Triage and Advice (MTS/TTA). Results were evaluated against a set of evaluation criteria.

**Results**

Implementation of MTS/TTA changed the response profile of emergency services in the Azores. There has been a reduction of high priority responses, and a corresponding increase in self-care and alternative services. Ability to respond to high acuity patients improved from 74.1% to 99.6%. Patient satisfaction remains high and there have been significant system benefits as a result of better resource utilisation. Audit has revealed high levels of compliance to clinical application of the system.

**Conclusions**

The impact on resource utilisation led to an improvement in the ability to respond to emergencies, generating system wide savings, both of which have the potential to provide clinical benefits overall. Further work is needed to evaluate the direct referral of patients into a fragmented primary care system.

## A16 Can CCTV improve the dispatcher’s situation awareness and their ability to lead the bystanders?

### Gitte Linderoth^1^*, Freddy Lippert^1^, Thea Palsgaard Møller^1^, Doris Østergaard^2^

#### ^1^Emergency Medical Services Copenhagen, University of Copenhagen, Copenhagen, Denmark; ^2^Copenhagen Academy for Medical Education and Simulation, Herlev, Denmark

##### **Correspondence:** Gitte Linderoth (gitte.linderoth@gmail.com)

**Background**

Emergency medical dispatchers’ identification of out-of-hospital cardiac arrest (OHCA) through emergency calls and provision of telephone assisted cardiopulmonary resuscitation (t-CPR) take place in a complex nonvisual work environment. We aimed at exploring emergency medical dispatchers’ perceived benefit with additional knowledge from CCTV in handling out-of-hospital cardiac arrest (OHCA).

**Materials and methods**

We performed an explorative interview study with dispatchers who handled an emergency call concerning OHCA captured on CCTV. We analysed the dispatchers’ perception of the bystander response and their reflections after seeing the CCTV. The interview participant first listened the to the emergency call and describe their perception of the scenario before seeing the CCTV recording. Afterwards, a semi-structured individual interview was conducted. Qualitative analysis based on thematic content analysis was used, with focus on the interval until the arrival of the ambulance.

**Results**

Based on 10 interviews, the main perceived benefits of seeing the CCTV were saved time, improved t-CPR and enhanced ability for the dispatcher to lead a team. Time could be saved because of faster identification, more focused questions and earlier information about the safety at the scene. The dispatcher could ensure the quality of CPR, see the bystanders and the physical position of the caller, correct misleading information and obtain the same and shared perception of the situation as the bystanders, easing communication.

**Conclusions**

The CCTV enhances the dispatcher situation awareness, which might save time and improve their ability to assist and lead the bystanders indicating that CCTV or use of smartphones could improve t-CPR.

## A17 Facilitating bystander CPR – a social anthropological study of behavioral change on the Danish island of Bornholm

### Grethe Thomas^1^*, Anne Møller Nielsen^2^, Gertrud Øllgaard^3^

#### ^1^TrygFonden, Virum, Denmark; ^2^Department of Anaesthesia, Centre of Head and Orthopaedics, Copenhagen University Hospital, Rigshospitalet, Copenhagen, Denmark; ^3^NIRAS, Allerød, Denmark

##### **Correspondence:** Grethe Thomas (gt@trygfonden.dk)

**Background**

Bystander CPR (bCPR) increases survival after OHCA. However, knowledge about what makes laypeople perform bCPR is missing.

The aim of this study is to create new insights into the social mechanisms that increase readiness to perform bCPR.

**Materials and methods**

From 2008-2010 an intervention was performed on Bornholm (Bornholm to the Rescue) with focus on education in BLS, implementation of AEDs and mass media focus on resuscitation, bCPR increased from 22% to 74% for witnessed OHCA.Three years after the intervention bCPR was 78%. To analyze this change a team of social anthropologists conducted fieldwork - observations of everyday situations and qualitative interviews with laypeople and healthcare professionals, journalists and other stakeholders.

**Results**

An essential finding is that domestication must take place to increase readiness to perform bCPR. Domestication refers to a socio-cultural process where new – technical, practical and moral – elements are incorporated into everyday life thereby laying the foundation for behavioral change. Central elements in this change were:

• A local initiator: anchored the project locally as a basis for support and commitment

• Local media: disseminated experiences with OHCA making bCPR relevant

• Courses at workplaces: embedded CPR skills in meaningful contexts

• MiniAnne®: an object to practice CPR skills and make the issue present in everyday life

• Public AEDs: a help in OHCA situations and a permanent reminder of one’s obligation to act

**Conclusions**

Bornholm to the rescue succeeded in domesticating these elements thereby enhancing many islanders’ readiness to act. A new readiness to perform bCPR was developed through processes of domestication.

## A18 Factors associated with survival from emergency medical service-witnessed out-of-hospital cardiac arrests: significance of early prehospital return of spontaneous circulation

### Hideo Inaba^1^*, Akira Yamashita^1,2^, Testuo Maeda^1^

#### ^1^Department of Emergency Medical Science, Kanazawa University Graduate School of Medicine, Kanazawa, Japan; ^2^Department of Cardiology, Noto General Hospital, Nanao, Japan

##### **Correspondence:** Hideo Inaba (mauriakoi@ybb.ne.jp)

**Background**

To determine the factors associated with prehospital return of spontaneous circulation (ROSC) and one-month neurologically favorable survival in emergency medical service (EMS)-witnessed out-of-hospital cardiac arrests (OHCAs).

**Materials and methods**

From the prospectively collected nationwide data on OHCAs between 2007 and 2012, complete dataset for 42,487 EMS-witnessed OHCAs without any prehospital involvement of physician was analyzed.

**Results**

Prehospital ROSC was recorded in 14.6% (6,224) of the cases and intensely associated with one-month neurologically favorable survival: 38.4% (2,387/6,224) in the cases with prehospital ROSC and 2.4% (871/36,263) without prehospital ROSC (OR; 95%CI, 25.3; 23.2–27.5). Furthermore, the survival rate was more augmented when the ROSC was obtained earlier after witness: 46.4% (1,931/4,158) with early (<7 min after witness) ROSC. Most (3,788, 91.1%) of early ROSC was induced by basic life support and fundamental airway management: 1,523 cases with defibrillation and 2,165 without defibrillation. Late prehospital ROSC was significantly associated with prehospital advanced life support (ALS) procedures. The best-fit models obtained by multiple logistic regression analysis disclosed that early prehospital ROSC (12.4; 10.9–14.2 with no prehospital ROSC as reference, 1.81; 1.58–2.07 with late prehospital ROSC as reference), in addition to EMS-performed defibrillation (4.01; 3.65–4.41), was significantly associated with overall one-month neurologically favorable survival from EMS-witnessed OHCAs and that prehospital ALS procedures was associated with poor one-month outcome (0.38; 0.34–0.44).

**Conclusions**

Standard basic life support and non-advanced airway management with or without defibrillation can yield early ROSC, which is potently associated with survival from EMS-witnessed OHCAs.

## A19 First aid training in Norway: an overview of prevalence, providers, and legislation

### Håkon Kvåle Bakke^1,2^*, Tine Steinvik^2^, Johan Angell^3,4^, Torben Wisborg^2,5,6^

#### ^1^Mo i Rana Hospital, Helgeland Hospital Trust, Mo i Rana, Norway; ^2^Anaesthesia and Critical Care Research Group, Faculty of Health Sciences, University of Tromsø, Tromsø, Norway; ^3^Lawyers Leiros & Olsen AS, Tromsø, Norway; ^4^Faculty of Law, University of Tromsø, Tromsø, Norway; ^5^Hammerfest Hospital, Department of Anaesthesiology and Intensive Care, Finnmark Health Trust, Hammerfest, Norway; ^6^Norwegian National Advisory Unit on Trauma, Division of Emergencies and Critical Care, Oslo University Hospital, Oslo, Norway

##### **Correspondence:** Håkon Kvåle Bakke (hakonkvalebakke@gmail.com)

**Background**

Bystander first aid can improve survival following out-of-hospital cardiac arrest or trauma. Thus, providing first aid education to laypersons leads to better outcomes. In this study we aimed to assess the prevalence of first aid training in the Norwegian populace, to describe the organisation of first aid training, and to examine the legislation concerning first aid training and provision in Norway.

**Materials and methods**

We conducted a telephone survey of 1,000 respondents who were representative of the Norwegian population. We also identified and interviewed entities that offered first aid training, and reviewed the legislation for laws and regulations governing first aid.

**Results**

Among the respondents, 90% had received first aid training, and 54% had undergone first aid training within the last 5 years. The workplace was the most common source of first aid training. Of the 43% who had been in a situation requiring first aid, 89% had provided first aid in that situation. We identified 192 organisations and enterprises offering first aid training, which mainly adhered to guidelines of the Norwegian Resuscitation and First Aid Councils. We identified four regulations governing first aid, although first aid was frequently mentioned in preparatory legal work.

**Conclusions**

A high proportion of the Norwegian population had first aid training, and interviewees reported high willingness to provide first aid. We identified several points that warrant further investigation, including the mode and content of first aid training, intent of legislation, and enactment of the first aid curriculum in the school system.

## A20 Guidance to bystanders in trauma: Emergency Medical Communication Centres strive to identify indicated first aid measures

### Håkon Kvåle Bakke^1,2^*, Tine Steinvik^2^, Håkon Ruud^3^, Torben Wisborg^2,4,5^

#### ^1^Mo i Rana Hospital, Helgeland Hospital Trust, Mo i Rana, Norway; ^2^Anaesthesia and Critical Care Research Group, Faculty of Health Sciences, University of Tromsø, Tromsø, Norway; ^3^Clinic of Emergency Medical Services, University Hospital of Northern Norway Harstad, UNN Hospital Trust, Harstad, Norway; ^4^Hammerfest Hospital, Department of Anaesthesiology and Intensive Care, Finnmark Health Trust, Hammerfest, Norway; ^5^Norwegian National Advisory Unit on Trauma, Division of Emergencies and Critical Care, Oslo University Hospital, Oslo, Norway

##### **Correspondence:** Håkon Kvåle Bakke **(**hakonkvalebakke@gmail.com)

**Background**

Emergency Medical Communication Centres (EMCC) dispatch and allocate ambulance resources, and provide guidance to on-scene bystanders providing first aid. We aimed to 1) evaluate whether dispatcher guidance improved bystander trauma first aid and 2) to evaluate if the dispatcher and the on-scene EMS crew found similar need for first aid measures.

**Materials and methods**

For 18 months the crew of the first ambulance responding to trauma calls assessed and documented the first aid performed by bystanders using a standard form. The audio recording of the corresponding telephone calls from the bystanders to the EMCC were later reviewed.

**Results**

A total of 311 trauma calls were included. Road traffic accidents and falls were the most common. The first aid measure free airway was found indicated by the first ambulance on scene in 26 patients, and EMCC advised to provide free airway for 62% (16/26) of these. CPR was indicated for 6 patients, and EMCC had given advice for 83% (5/6) of these. Hypothermia prevention was indicated for 179 patients, and EMCC had given advice for 30% (54/179) of these. We found no correlation between guidance from EMCC being given and whether the measure in question had been carried out correctly at the scene of accident (p = 0.3-0.6)

**Conclusions**

The Emergency Medical Communication Centres have trouble correctly identifying the trauma patients in need of several first aid measures. Guidance from EMCC did not seem to affect the quality of first aid on scene.

## A21 The first resuscitation team: training elderly for 30:2 CPR with dispatcher assistance. A pilot test

### Ingunn Anda Haug^1^*, Tonje S. Birkenes^1^, Helge Myklebust^1^, Jo Kramer-Johansen^2^

#### ^1^Laerdal Medical, Stavanger, Norway; ^2^Norwegian National Advisory Unit on Prehospital Emergency Medicine (NAKOS), Oslo, Norway

##### **Correspondence:** Ingunn Anda Haug (ingunn.haug@laerdal.com)

**Background**

Guidelines 2015 recommends 30:2 for trained CPR providers. Mouth to mouth is difficult to perform even for trained providers, and telephone CPR can improve CPR quality. The main objective of this pilot study was to test if trained elderly lay people can perform quality compressions and ventilations (30:2) with dispatcher assistance.

**Materials and methods**

Elderly lay people were first trained in T-CPR with chest compression only, and later in T-CPR with 30:2 CPR. A simulation test was performed before (pretest) and after (posttest) the 30:2 training. During the simulation, participants received dispatcher instructions to perform 230s of 30:2 CPR with continuous coaching. CPR performance was recorded using ResusciAnne (Laerdal Medical). Based on median ventilation volume provided in the pretest, participants were split to Group 1 (>600ml); Group 2 (200-600ml); Group 3 (<200ml). CPR performance data was analyzed from pretest to posttest.

**Results**

Twenty-nine people (age 57-83) participated. Pretest placed 20/29 participants in Group 1, 1/29 in Group 2 and 8/29 in Group 3, improving to 12/29, 15/29 and 2/29 in posttest, respectively. Group 1 improved the ventilation volume from median 893 to 623 ml (p < 0.01), Group 2 maintained a proper volume, and Group 3 improved volume from median 0 to 413 (p =0.03). Overall, time spent on each ventilation interval improved from mean 12 to 10 s (p < 0.01). The total number of compression improved from mean 227 to 251 (p < 0.01).

**Conclusions**

In this pilot study, trained elderly lay people performed quality CPR 30:2 with dispatcher instructions and continuous coaching.

## A22 Quality of life after trauma before and after implementation of physician-staffed helicopter emergency medical services

### Kamilia S. Funder^1^*, Lars S. Rasmussen^1^, Rasmus Hesselfeldt^2^, Volkert Siersma^3^, Nicolai Lohse^1^, Asger Sonne^1^, Sandra Wulffeld^1^, Jacob Steinmetz^1^

#### ^1^Department of Anaesthesia, Centre of Head and Orthopaedics, Copenhagen University Hospital Rigshospitalet, Copenhagen, Denmark; ^2^Department of Neuroanaesthesiology, Neuroscience Centre, Copenhagen University Hospital, Rigshospitalet, Copenhagen, Denmark; ^3^The Research Unit for General Practice and Section of General Practice, Department of Public Health, University of Copenhagen, Copenhagen, Denmark

##### **Correspondence:** Kamilia S. Funder (milafun@hotmail.com)

**Background**

Implementation of Helicopter Emergency Medical Services (HEMS) in Denmark in 2010 was associated with lower 30-day mortality in severely injured trauma patients. We aimed to investigate if HEMS implementation was associated with improved quality of life (QoL) among survivors.

**Materials and methods**

A prospective, observational study including trauma patients who survived at least 3 years. A 5-month period prior to HEMS implementation (pre-HEMS) was compared with the first 12 months after implementation (post-HEMS). QoL was assessed 4.5 years after trauma by the self-reported Short Form-36 (SF-36) questionnaire. SF-36 scores are standardized to a mean of 50 (SD = 10) in a given background population, with higher scores indicating higher level of functioning.

**Results**

Of the total 1976 patients, 1162 were alive and with known contact information. We obtained contact to 659 (57%), and 469 (40%) returned the SF-36 questionnaire (n = 131 pre-HEMS and n = 338 post-HEMS). Older patients, women, and patients with trauma in the post-HEMS period were most likely to respond. Median Physical Component Summary score was 50.7 (Interquartile Range 40.9-58.1) pre-HEMS and 51.9 (42.1-58.0) post-HEMS (p = 0.66). Median Mental Component Summary score was 54.7 (46.1-59.7) pre-HEMS and 52.4 (41.8-58.8) post-HEMS (p = 0.18). No significant association between QoL and observational period was found in a multivariable analysis.

**Conclusions**

We found no significant changes in QoL among trauma survivors after implementation of HEMS.

## A23 No beneficial effect of a physician-manned helicopter on patients bound for percutaneous coronary intervention

### Kamilia S. Funder^1^*, Lars S. Rasmussen^1^, Nicolai Lohse^1^, Rasmus Hesselfeldt^2^, Volkert Siersma^3^, Frants Pedersen^4^, Ole M. Hendriksen^5^, Freddy K. Lippert^6^, Jacob Steinmetz^1^

#### ^1^Department of Anaesthesia, Centre of Head and Orthopaedics, Copenhagen University Hospital Rigshospitalet, Copenhagen, Denmark; ^2^Department of Neuroanaesthesiology, Neuroscience Centre, Copenhagen University Hospital, Rigshospitalet, Copenhagen, Denmark; ^3^The Research Unit for General Practice and Section of General Practice, Department of Public Health, University of Copenhagen, Copenhagen, Denmark; ^4^Department of Cardiology, The Heart Centre, Copenhagen University Hospital, Rigshospitalet, Copenhagen, Denmark; ^5^Prehospital centre, Region Zealand, Denmark; ^6^Emergency Medical Services Copenhagen, University of Copenhagen, Copenhagen, Denmark

##### **Correspondence:** Kamilia S. Funder (milafun@hotmail.com)

**Background**

The first Danish Helicopter Emergency Medical Service (HEMS) was introduced in 2010. The implementation was associated with reduced time from medical contact to final treatment for patients with ST elevation myocardial infarction. We aimed to investigate the effects of HEMS on mortality and labour market affiliation for patients admitted for primary Percutaneous Coronary Intervention (pPCI).

**Materials and methods**

A prospective, observational, study with follow-up up to 5.5 years. We compared patients transported by HEMS in a 36-month period with patients transported by Ground Emergency Medical Service (GEMS) in a 40-month period. Analyses were adjusted for sex, age, resuscitated cardiac arrest, inter-hospital transfer, and acute cardiac failure.

**Results**

We included 1604 patients of whom 514 were eligible for labour market analyses. 30-day mortality was 5.0% (HEMS) vs. 6.2% (GEMS); Odds Ratio (OR) = 0.82 (95% CI 0.44-1.51; p = 0.52). Hazard Ratio (HR) for long-term mortality was 0.83 (0.62-1.11; p = 0.21). A higher, but insignificant difference in rate of involuntary early retirement was found in HEMS-patients; HR = 1.46 (0.31-6.88; p = 0.63). The proportion of patients on social transfer payments more than half the follow-up time was 22.1% vs. 21.2%, OR = 1.10 (0.64-1.90; p = 0.73), and the prevalence of reduced work ability was 23.0% vs. 25.9%, OR = 0.91 (0.52-1.60; P = 0.75). Time (hours) from diagnostic electrocardiogram to PCI centre arrival was 1.30 (IQR 0.93-1.78) vs. 1.18 (1.00-1.43) for GEMS and HEMS patients, respectively (p = 0.004).

**Conclusions**

No beneficial effect of HEMS could be demonstrated in patients admitted for pPCI as there were no significant differences in mortality, premature labour market exit, or work ability.

## A24 Validation of criteria for trauma center resources need in emergency medical services-treated severe trauma patients

### Kim Sol-A*, Sand Do Shin, Kyungwon Lee, Eui Jung Lee, Young Sun Ro, Ki Jeong Hong, Yu Jin Kim, Joo Jeong, Park Jeong Ho

#### Laboratory of Emergency Medical Service, Bio-medical Research Institute, Seoul National University Hospital, Seoul, South Korea

##### **Correspondence:** Kim Sol-A (arendt75@gmail.com)

**Background**

This study aimed to validate criteria for trauma center (TC) need and compare with validity of the ISS > 15 tool, which is a commonly used measure of outcome in TC need research.

**Materials and methods**

Emergency medical services (EMS)-treated severe trauma (ST) patients who had abnormal revised trauma score in the field or were triaged positive by the field triage protocol in 2013 were analyzed. Patients who had prehospital cardiac arrest, were transferred out to other hospitals, or had unknown ISS were excluded. ISS > 15 was defined as severe trauma. Criteria for TC need were defined as any use of TC resource (advanced airway management (<4hrs), thoracotomy (<48hrs), interventional radiology (<4hrs), non-orthopedic operation (<24hrs), and admission for spinal cord injury). Primary endpoint was in-hospital death.

**Results**

A total of 14,352 adult EMS-ST patients was identified. 9,435 patients were included in the analysis after excluding patients. About 18.4% had ISS > 15, and 22.8% required use of TC resource. Rate of in-hospital death was 10.6%. Sensitivity and specificity of the ISS > 15 group were 60.6% (95% CI, 57.5% ~ 63.6%) and 86.6% (95% CI, 85.9% ~ 87.3%), respectively, for in-hospital death. For the criteria for TC need, sensitivity and specificity for in-hospital death were 88% (95% CI, 85.9% ~ 90%) and 85% (95% CI, 84.2% ~ 85.8%), respectively.

**Conclusions**

Criteria for TC need are relatively more sensitive than ISS > 15 tool for identifying in-hospital death in patients triaged with severe trauma in prehospital settings.

## A25 A philosophical approach to the ethics of terminating prehospital resuscitative efforts at the scene

### Lars Grassmé Binderup^1^, Søren Mikkelsen^1^*, Caroline Schaffalitzky de Muckadell^2^, Hans Morten Lossius^3^, Palle Toft^1^, Annmarie Touborg Lassen^4^

#### ^1^Mobile Emergency Care Unit, Deparment. Anaesthesiology and Intensive Care Med. V, Odense University Hospital, Odense, Denmark; ^2^Philosophy, Department for the Study of Culture, University of Southern Denmark, Odense, Denmark^3^Prehospital Critical Care, Stavanger University, Stavanger, ; Norway; ^4^Emergency Medicine Research Unit Odense University Hospital, Odense, Denmark

##### **Correspondence:** Søren Mikkelsen (Soeren.mikkelsen@rsyd.dk)

**Background**

In the Intensive Care Unit or Emergency Department, discussions on the ethical aspects of the question whether to resuscitate or not often take place between several physicians. Prehospitally, however, most often only one physician is present. This often leaves the physician with an ethical dilemma to be tackled without recourse to collegial sparring and advice: Should one continue resuscitative efforts that from the onset are considered futile or should one terminate or refrain from resuscitation on the assumption that the patient is beyond salvageability. This is a study of documentation of prehospital ethical considerations.

**Materials and methods**

The study is based on the discharge summaries relating to all patients declared dead without displaying certain signs of death by the Mobile Emergency Care Unit in Odense Denmark in the period 2010 to 2014. Medical records were reviewed manually to identify explicit ethical or philosophical considerations pertaining to the decision not to resuscitate.

**Results**

2,227 patients were declared dead at the scene or admitted to hospital following resuscitation. 952 patients displayed signs of death and were excluded. 1,275 patients entered the study. Explicit ethical considerations were described in 37 cases; five in patients resuscitated and admitted to hospital; 15 in patients attempted resuscitated in vain. In 17 patients not resuscitated, ethical considerations were documented.

**Conclusions**

A significant absence of documented explicit ethical considerations pertaining to important life and death decisions has been demonstrated. It may be appropriate to introduce mandatory documentation of ethical considerations when refraining from resuscitation of potentially salvageable patients prehospitally.

## A26 Identifying pre-hospital factors which influence outcome for major trauma patients in a regional trauma network: an exploratory study

### Lee Thompson^1^*, Michael Hill^2^

#### ^1^North East Ambulance Service, Newcastle upon Tyne, UK; ^2^Northumbria University, Newcastle upon Tyne, UK

##### **Correspondence:** Lee Thompson (lee.thompson@neas.nhs.uk)

**Background**

Major trauma is often life threatening/changing and the leading cause of death in the UK for adults aged < 45 years. This study aimed to identify pre-hospital factors influencing patient outcomes for major trauma within a Trauma Network.

**Materials and methods**

Secondary data analysis of combined pre-hospital audit data and patient outcome data from the Trauma Audit Research Network (n = 1,033) was undertaken. Variables included mechanism of injury, age, physiology, timings and skill mix. Outcome measures included Mortality data and Glasgow Outcome Scale.

**Results**

GCS proved a significant predictor of mortality (p < 0.00). Systolic BP < 90 mm Hg. was associated with increased mortality (p < 0.004) and poorer morbidity (p < 0.021). Respiration rate < 14/minute was also predictive of morbidity (p < 0.03) and mortality (p < 0.00). Response times > 10 minutes (P < 0.031) and increasing age were significantly associated with poorer outcomes. Attendance of a Doctor was significantly associated with increased mortality (p < 0.036) perhaps validating existing despatching practices. Predictors of positive outcomes included the presence of a Doctor when on-scene time < 50 minutes (p < 0.015) and crew arrival on-scene < 10 minutes (p < 0.046).

**Conclusions**

Findings validate GCS, BP and Respiratory Rates as valid triggers for transport to a MTC. Analysis of interactions between arrival and on-scene times, skill mix and age demand further exploration but tentatively validate the ‘Golden Hour’ concept and suggest a potential ‘load and go and play on the way’ approach.

## A27 Prehospital diagnostics of traumatic brain injury: to save both time and brain

### Maren Ranhoff Hov^1^*, Thomas Lindner^1^, Eirik Franer^2^, Andreas Monstad^2^, Christian G Lund^1^

#### ^1^The Norwegian Air Ambulance Foundation, Grålum, Norway; ^2^Østfold Hospital, Grålum, Norway

##### **Correspondence:** Maren Ranhoff Hov (maren.ranhoff.hov@norskluftambulanse.no)

**Background**

To make a prehospital cerebral radiological diagnosis in traumatic brain injury (TBI). TBI is a leading cause of death and severe disability in not at least young people, and time to definite neurosurgical treatment is prognostic.

**Materials and methods**

Since October 2014 the Norwegian Air Ambulance Foundation has run a “Mobile Brain Unit (MBU)” ambulance study in the county of Østfold, Norway. The MBU is equipped with a CT scanner and manned with an anesthesiologist, an intensive care nurse and a paramedic, a staff used by the national operating Air Ambulance system. Patients with symptoms of acute stroke or a history of TBI are included in the study. The anesthesiologist interprets all cerebral CT scans before consulting the local radiological department by teleradiology.

**Results**

Two TBI patients have been diagnosed in the MBU with epidural hematoma and traumatic subarachnoid hemorrhage and epidural hematoma, respectively. Both were transported directly from the trauma scene to the regional neurosurgical department, avoiding the local hospital. “Ambulance door to diagnostic time” was mean 11 minutes. Mean total time from trauma to neurosurgery care was 1 hour and 26 minutes. Estimated time saved when avoiding the local hospital has been approximately 3 hours in each case.

**Conclusions**

The MBU concept with prehospital CT diagnostics will reduce time delay to neurosurgical care in TBI, which in many cases very likely will give a better outcome.

## A28 Paediatric out of hospital cardiac arrest: comparison of manual and automatic heart rhythm analysis – a pilot study

### Martin Betzer^1^*, Rasmus Malmberg Lyngby^2^

#### ^1^Prehospital Centre, Region Zealand, Denmark; ^2^Emergency Medical Services Copenhagen, Capital Region of Denmark, Denmark

##### **Correspondence:** Martin Betzer (martin.betzer@gmail.com)

**Background**

In paediatric out of hospital cardiac arrest in Denmark, the shock/no shock rhythm analysis is done manually by ambulance person (AP). The aim of this study is to explore the accuracy of, and time spend on this manual rhythm analysis compared to that of an automatic external defibrillator (AED) used in adults.

**Materials and methods**

Out of an invited group of 39 PT, 14 participated in the study. All participants were presented the same 20 cardiac arrest heart rhythms by online questionnaire (5xVF, 3xVT, 2× slow VT, 5xAS, 5xPEA). Each rhythm was presented as a 5-year-old in cardiac arrest. The participants were asked to classify the rhythms shockable/non-shockable. The AED was presented the same 20 heart rhythms for comparison. In both groups, analysis time was obtained.

**Results**

The average analysis time for the AP was 6.4 seconds per rhythm with an accuracy of 93%. The AED’s average analysis time was 6.7 seconds with a 100% analysis accuracy.

**Conclusions**

AP are capable of analysing the heart rhythm in paediatric cardiac arrest faster than an AED. However, the AED analysis is more accurate. There is a need for larger scale study to further explore this matter.

## A29 Point-of-care analysis of potassium from intraosseous samples

### Milla Jousi*, Jouni Nurmi

#### Department of Emergency Medicine, Helsinki University Hospital and University of Helsinki, Helsinki, Finland

##### **Correspondence:** Milla Jousi (milla.jousi@gmail.com)

**Background**

Diagnosing and treating reversible causes of cardiac arrest during resuscitation, such as hyperkalemia, is an integral part of the treatment protocol. Intraosseous (IO) access aided by power-driven devices has become a widely used method of vascular access during CPR and facilitates blood sampling for point-of-care (POC) analysis. The aim was to investigate whether POC potassium levels of intraosseous and arterial blood correspond with sufficient precision so that IO samples could be used for clinical decision-making.

**Materials and methods**

This was an observational study comparing POC results of potassium from IO and arterial blood of 31 healthy volunteers. Venous samples were analyzed as controls. Intraosseous access with EZ-IO® was obtained to the proximal tibia. Blood samples were analyzed with a point-of-care device (i-STAT® handheld). Two consecutive intraosseous samples were analyzed from each patient to evaluate the effect of waste blood (IO1 of initial 0.5 ml and IO2 after 2 ml of waste blood). Test results were compared with Wilcoxon matched-pairs signed rank test. The ethical committee had approved the study protocol.

**Results**

59 out of 62 IO-samples (95%) were analyzed successfully. Potassium values of venous and arterial samples did not differ significantly (median difference 0.1 mmol/l [IQR 0.00 – 0.20], P = 0.88). Both IO1 and IO2 were significantly higher than arterial values (median difference 2.2 mmol/l [IQR 1.5 – 7.1], P < 0.0001 and 3.4 mmol/l [IQR 2.4 – 4.2], P < 0.0001, respectively).

**Conclusions**

Intraosseous potassium values are significantly higher than arterial values and cannot be recommended for clinical decision-making.

## A30 Complications during transfer of critically ill patients

### Nanna Kruse^1^*, Charlotte Barfod^2^

#### ^1^Department of Aneasthesia and Intensive Care, Hillerød Hospital, Hillerød, Denmark; ^2^Emergency Medical Services Copenhagen, University of Copenhagen, Copenhagen Denmark

##### **Correspondence:** Nanna Kruse (nannakruse1@gmail.com)

**Background**

Transfer of critically ill patients between hospitals is a high risk procedure. Therefore the benefits of transferring the patients should exceed the possible threats. In this study, we registered complications during interhospital transfer of critically ill patients.

**Materials and methods**

We included all interhospital transfers escorted by a physician specialized in advanced prehospital care during a 3 months period in the Capital Region in Denmark. The variables registered were time and date, patient age and diagnosis and the presence of clinical or technical complications. Clinical complications were defined as accidental extubation, need for intubation, desaturation (<85%), cardiac arrest, hypotension (systolic blood pressure below 80 mmHg) or a decrease in Glasgow Coma Score (GCS) 2 points or more. Technical complications covered malfunction of technical devices.

**Results**

A total of 285 transfers were done in the three months period. A total of 41 patients (14%) experienced complications during transfer. 60% were handled by the escorting physician before leaving the hospital, 40% were handled on the road. Cardiac arrests occurred in 4 patients, 2 cases before transfer and 2 during transportation. In 8 patients hypotension was registered. A number of 6 patients needed intubation, 5 before and 1 during transfer. In 2 patients GCS declined 2 points or more. In 6 transfers the crew experienced technical problems.

**Conclusions**

Potentially serious complications are frequent during transfer of critically ill patients. We therefore suggest the crew transferring the patient should include an experienced physician specialized in advanced prehospital care.

## A31 “Unclear Problem” in criteria-based dispatch – what is the problem?

### Nikolaj Raaber*, Morten T. Bøtker, Sophie-Charlott Seidenfaden, Ingunn S. Riddervold

#### Department of Research and Development, Prehospital Emergency Medical Services, Central Denmark Region, Aarhus, Denmark

##### **Correspondence:** Nikolaj Raaber (nikoraab@rm.dk)

**Background**

In Denmark, ambulance dispatch is criteria-based. When the dispatching health professional is not able to determine the main cause of contact, the dispatch criterion “Unclear Problem” is used. Early recognition of time critical conditions defined by the First Hour Quintet (FHQ) covering trauma, respiratory failure, stroke, chest pain and cardiac arrest, is essential. The aim of this study was to describe ICD-10-chapter and FHQ-diagnosis in “Unclear Problem” patients and the associated mortality.

**Materials and methods**

Population-based follow-up study of patients calling 112 in the Central Denmark Region assigned dispatch criteria “Unclear Problem” from October 1 2011- December 31 2014. ICD-10 diagnoses were retrieved from the Danish National Patient Registry and vital status from the Danish Civil Registration system.

**Results**

“Unclear Problem” accounted for 6,402/75,696 (8.5%) of 112-contacts. When categorized into main ICD-10 chapters, the five most frequent groups were; “C19 - Injury, poisoning and external causes” (28%), “C21 - Symptoms and encounters for diagnoses” (24%), “C18 - Symptoms and signs not elsewhere classified” (15%), “C9 Diseases of the circulatory system” (10%) and “C10 Disease of the respiratory system” (3.7%). Frequency of all other chapters < 3%. 396/6,402 (6.2%) were diagnosed with one of the FHQ-diagnoses. Overall 30-day mortality rate for “Unclear Problem” patients was 3.5% (95% CI 3.1 – 4.0) and FHQ-patient mortality was 9.4 (95% CI 6.7 – 13).

**Conclusions**

“Unclear Problem” covers a wide variety of diagnosis. Patients subsequently diagnosed with FHQ-diagnosis have a higher mortality rate than other patients covered by the “Unclear Problem” dispatch criterion.

## A32 Prevalence and predictors of burnout in Australian paramedics

### Paul Simpson^1^*, Liz Thyer^1^, Ben van Nugteren^2^

#### ^1^Western Sydney University, Sydney, Australia; ^2^University of Johannesburg, Johannesburg, South Africa

##### **Correspondence:** Paul Simpson (p.simpson@westernsydney.edu.au)

**Background**

Work ‘burnout’ is a significant issue in paramedicine, with evidence suggesting paramedics have the highest incidence of burnout of any researched health profession. Burnout has been linked to decreased job satisfaction, increased absenteeism, low morale, decreased job retention, poor patient care and decreased emotional and physical wellbeing. The aims of this study were to 1) investigate the prevalence of burnout in paramedics, and 2) identify demographic predictors of burnout.

**Materials and methods**

An online survey of Australian paramedics was conducted using a national convenience sample. Potential respondents were recruited using social media, print media and ‘snow-balling’, as no single point of access to the paramedic population was available. Self-reported burnout was measured using a validated survey instrument, the Copenhagen Burnout Inventory (CBI). The tool provides a continuous measure of burnout, and allows creation of a dichotomous outcome of burnout status (yes or no). Multiple logistic regression modelling was used to identify demographic factors (age, gender, years of service, rurality, marital status) predictive of burnout.

**Results**

960 paramedics responded to the survey. The overall prevalence of total burnout was 56%. Working in major cities was associated with greater odds of burnout compared to rural locations (OR 2.7; 95%CI 1.3-5.3), as was increasing length of service (OR 3.7 for 15-19 years of service compared to 0-5; 95%CI 2.3-6.1). Males had 33% lower odds of burnout (OR 0.67; 95%CI 0.5-0.9).

**Conclusions**

Prevalence of burnout was high in this paramedic population. Qualitative research is recommended to better understand the identified demographic predictors of total burnout.

## A33 Traumatic brain injury – cause of emergency medical calls

### Sophie-Charlott Seidenfaden^1^*, Ingunn S. Riddervold^1^, Hans Kirkegaard^2^, Niels Juul^3^, Morten T. Bøtker^1^

#### ^1^Department of Research and Development, Prehospital Emergency Medical Services, Central Denmark Region, Aarhus, Denmark; ^2^Research Centre for Emergency Medicine, Aarhus University Hospital, Aarhus, Denmark; ^3^Department of Neuro Anaesthesiology, Aarhus University Hospital, Aarhus, Denmark

##### **Correspondence:** Sophie-Charlott Seidenfaden (soseid@rm.dk)

**Background**

Traumatic brain injury (TBI) is the leading cause of death in children and young adults. In Denmark, ambulance dispatch is criteria-based depending on main symptom or incident, but no separate dispatch criteria exist for head trauma or TBI. The aim of this study was to investigate which dispatch criteria are assigned to TBI patients and to examine 30-day mortality in patients suffering TBI.

**Materials and methods**

Population-based follow-up study of patients calling 112 in the Central Denmark Region and subsequently diagnosed with TBI according to ICD-10 codes registered in the Danish National Patient Registry from October 1 2011 to December 31 2014. We predefined 7 of 37 dispatch criteria to expectedly be assigned to TBI patients.

**Results**

Of 75,696 112-calls, 1427 patients were subsequently diagnosed with TBI (1.9%). The five most frequent dispatch criteria were; “Accidents” 47%, “Traffic accident” 19%, “Minor injuries” 10%, “Impaired consciousness - paralyses” 6%, “Violence” 5%. In 22% of TBI patients, other than the expected dispatch criteria were assigned. 30-day mortality rate in TBI patients was 1.6% (95% CI 1.1-2.4%). In patients with confirmed intracranial lesions (bleeding/fracture, n = 229) 30-day mortality was 10% (95% CI 6.8-15). Of these patients, 14 of 229 (6%) did not receive an ambulance at initial 112-call.

**Conclusions**

Patients suffering TBI are assigned very different dispatch criteria. One in five were assigned criteria not generally associated with TBI. Modifications to the dispatch criteria may improve dispatch for TBI patients.

## A34 General Practitioners requesting emergency response - an evaluation of the communication with the medically trained personal at the dispatch central in the Region of Southern Denmark

### Stine T. Zwisler^1,2^, Camilla Rønnov^3^*, Hanne B. Mieritz^1^, Søren Mikkelsen^4,1^, Gitte Jørgensen^1,2^

#### ^1^Department of Anesthesiology and Intensive Care, Odense University Hospital, Odense, Denmark; ^2^Dispatch Central, Region of Southern Denmark, Odense, Denmark; ^3^University of Southern Denmark, Odense, Denmark; ^4^Mobile Emergency Care Unit Region of Southern Denmark, Denmark

##### **Correspondence:** Camilla Rønnov (camillar@me.com)

**Background**

When general practitioners (GPs) in Region of Southern Denmark need emergency response units they call the dispatch central, where medically trained personal (MTP) evaluates and prioritizes the response needed.

**Materials and methods**

This retrospective study is based on calls where GPs request emergency response including ambulance and sometimes the mobile emergency care unit (MECU). Calls were sought in the database containing all calls to the dispatch central. Inclusion period was two representative months, June and December, of 2014.1507 calls were evaluated according to specific parameters, and analysed using Eric Berner’s model for transactional analysis, by a medical student. Calls with communication issues and insufficient information was also evaluated by an anaesthesiologist.

**Results**

There were communicative problems in less than 2% of the calls. This occurred frequently when a nurse or secretary called instead of the GP, which they did in 4% of the calls. In 75% of calls with communication problems, transactional analysis showed that the MTPs talked in a condescending manner to the caller. GPs demanded MECU in 1% without symptoms meeting the criteria. GPs did not have enough information to validate requests for MECU in 5% of calls. MTP did not ask enough questions to decide if a MECU should be dispatched in 13% of calls.

**Conclusions**

2% of the calls revealed communicative problems, mostly between MTP and the GPs substitute caller.

## A35 Feasibility of cerebral near infrared spectroscopy monitoring in prehospital anesthesia

### Susanne Ångerman-Haasmaa*, Sami Länkimäki, Jouni Nurmi

#### Department of Emergency Medicine, Helsinki University Hospital and University of Helsinki, Helsinki, Finland

##### **Correspondence:** Susanne Ångerman-Haasmaa (angersu@icloud.com)

**Background**

Near infrared spectroscopy (NIRS) provides a non-invasive measure of cerebral tissue oxygenation and can be useful during anesthesia when oxygenation is threatened. Feasibility of this method has not been properly evaluated in prehospital setting. The aim of the study was to determine the feasibility of NIRS monitoring during prehospital anesthesia.

**Materials and methods**

This was a prospective observational study and the protocol was approved by the ethical committee. Five HEMS-physicians recruited 32 adult patients who underwent prehospital anesthesia. NIRS monitoring (Nonin SenSmart X-100) was initiated before induction of anesthesia (baseline) and continued until arriving to hospital. The regional oxygen saturation (rSO_2_) values were analyzed afterwards.

**Results**

The age of the patients was 54 ± 16 years (mean ± SD) and 22 (69%) of them were male. The indications for prehospital anesthesia were neurological reasons 14 (44%), intoxications 7 (22%), traumatic brain injury 6 (19%) and cardiac arrest with ROSC 5 (16%). The NIRS monitoring was successful in 29 of 32 cases (91%; 95% CI 75 to 98%). Of the unsuccessful cases, two had short interruptions in the recording and one patient could not be monitored at all due to poor probe-skin contact. The duration of the monitoring was 45 ± 16 minutes. The baseline rSO_2_ were 73 ± 15% and 76 ± 14% on right and left hemispheres, respectively. The lowest rSO_2_ was 10 ± 9 (range 0-34) below and peak rSO_2_ 13 ± 10 (range 2 to 45) above the baseline.

**Conclusions**

NIRS-monitoring is feasible during prehospital anesthesia and substantial changes in rSO_2_ are observed in some of the patients.

## A36 Patients declared dead at the prehospital scene

### Søren Mikkelsen^1^*, Hans Morten Lossius^2^, Palle Toft^1^, Annmarie Touborg Lassen^3^

#### ^1^Mobile Emergency Care Unit, Department Anaesthesiology and Intensive Care Medecine, Odense University Hospital, Odense, Denmark; ^2^Prehospital Critical Care, Stavanger University, Stavanger, Norway; ^3^Emergency Medicine Research Unit, Odense University Hospital, Odense, Denmark

##### **Correspondence:** Søren Mikkelsen (Soeren.mikkelsen@rsyd.dk)

**Background**

The purpose of this study was to describe patients that are declared dead prehospitally by the Mobil Emergency Care Unit (MECU) physician despite the lack of reliable signs of death, and further to describe parameters associated to the physician decision to refrain from resuscitation.

**Materials and methods**

We performed a retrospective study of each emergency run by the MECU in Odense Denmark from 2010 to 2014 were the patient was declared dead without reliable signs of death or when the patient was attempted resuscitated. Data were collected electronically and by manual chart review.

**Results**

2,227 patients were declared dead at the scene or admitted to hospital following resuscitation. 952 patients displayed reliable signs of death on scene and were excluded from the study. The MECU physician declared 633 patients dead on scene without initiating resuscitative attempts and 338 patients were declared dead after resuscitative efforts. 304 patients were admitted to hospital following or during resuscitation. The 633 patients not attempted resuscitated were older, had a higher incidence of non-shockable cardiac rhythm, demonstrated fixed pupils to a higher extent, co-suffered more often a chronic disease including malignancy and were more prone to live in nursery homes than patients attempted resuscitated.

**Conclusions**

The MECU physicians often base the decision of not initiating resuscitative attempts on specific clinical findings other than reliable signs of death, as well as information regarding patient co-morbidity. Our findings may have a potential for deducting do-not-resuscitate algorithms for non-physician prehospital personnel.

## A37 Recognition of out-of-hospital cardiac arrest during emergency calls – a systematic review of observational studies

### Søren Viereck^1^*, Thea Palsgaard Møller^1^, Josephine Philip Rothman^2^, Fredrik Folke^1^, Freddy K. Lippert^1^

#### ^1^Emergency Medical Services, University of Copenhagen, Copenhagen, Denmark; ^2^Center for Perioperative Optimization, Department of Surgery, Herlev Hospital, University of Copenhagen, Copenhagen, Denmark

##### **Correspondence:** Søren Viereck (soeren.viereck@regionh.dk)

**Background**

Survival from Out-of-Hospital Cardiac Arrest (OHCA) rarely exceeds 10%, and highly depends on cardiopulmonary resuscitation (CPR) and defibrillation by an automated external defibrillator (AED). Recognition of OHCA by the emergency medical dispatcher (EMD) during emergency calls is a prerequisite for CPR and defibrillation. The EMD can provide telephone assisted CPR and refer the bystander to the nearest AED while dispatching an ambulance. It is shown that recognition of OHCA can increase survival 3-fold. We studied how accurately OHCA is recognized by EMD’s during emergency calls.

**Materials and methods**

A systematic review of English observational studies reporting the proportion of clinically confirmed OHCA’s recognized during emergency calls was performed. We searched MEDLINE, Embase and the Cochrane Library. Information on study setting, sensitivity and positive predictive value were extracted.

**Results**

In total, 3,180 studies were screened for eligibility. We included 15 studies from 16 EMS systems in 12 different countries, reporting a total of 6,955 patients. The studies reported a median incidence of 35.3 OHCA’s pr. 100,000 inhabitants pr. year (range: 6.1-129.3). The median sensitivity of OHCA recognition was 73.9% (range: 14.1%-96.9%). The median positive predictive value was 67.4% (range: 58.4%-97.9%). Selection of study-population and the definition of “recognized OHCA” varied greatly among studies.

**Conclusions**

Recognition of OHCA is essential for early CPR and the use of AED’s, thereby for survival. Few studies report recognition and the results are not reported in a uniform way. This challenges identification of best practice and benchmarking via this important performance indicator for quality.

## A38 Survey assessing patients' interest and opinion in receiving health information and education as part of their emergency department admission

### Teodora Filipescu^1^*, Alasdair Gray^1,2^

#### ^1^University of Edinburgh, Edinburgh, Scotland; ^2^Emergency Medicine NHS Lothian, Edinburgh, Scotland

##### **Correspondence:** Teodora Filipescu (teodora.filipescu@gmail.com)

**Background**

Patients suffering from potentially preventable conditions often present to Emergency Departments (ED). The time they spend waiting for medical attention could be used to deliver preventive health education, but patients’ needs and preferences must first be taken into consideration. The project aimed to find out whether ED patients want to receive general health education, and if so, how can this service be designed to best fit their needs.

**Materials and methods**

102 ED patients presenting to a large Scottish hospital were surveyed on their health topics interests and preferred educational delivery methods. Socio-demographic and some clinical data were collected for participant characterization. All ethical approvals were obtained prior to the beginning of the study.

**Results**

43% of respondents wanted health education in ED. Chronic diseases, cancer and stress were the most popular health topics. In contrast, traffic safety, safe sex and injury prevention were a low priority. Direct discussion with a consultant or specialist nurse and leaflets were the preferred delivery methods. Discussion This is the first study of this nature conducted in UK. Our results match those of two large US studies and raise questions on patients’ lack of interest in injury prevention and on the current trends in ED Public Health studies, which seem to be desynchronised with patients’ educational priorities.

**Conclusions**

Nearly half of patients welcome health education in a critical moment. With clinician and patient input alike, the ED could become an appropriate environment for effective ill-health prevention to a high-risk, yet interested audience.

## A39 Identification of critical illness in the prehospital setting

### Teresa A Williams^1,2,3,7^*, Kwok M. Ho^4,5^, Hideo Tohira^1^, Daniel Fatovich^6^, Deon Brink^2^, Paul Bailey^2^, Gavin D. Perkins^8,9^, Judith Finn^1^

#### ^1^Prehospital Resuscitation and Emergency Care Research Unit, Curtin University, Perth, Australia; ^2^St John Ambulance-Western Australia, Perth, Australia; ^3^Royal Perth Hospital, Perth, Australia; ^4^Intensive Care Unit, Royal Perth Hospital, Perth, Australia; ^5^School of Population Health, The University of Western Australia, Perth, Australia; ^6^Emergency Medicine, Royal Perth Hospital, Perth, Australia; ^7^Centre for Clinical Research in Emergency Medicine, Harry Perkins Institute of Medical Research, Nedlands, Australia; Critical Care Medicine, Warwick Medical School, University of Warwick, Coventry, UK; Heart of England NHS Foundation Trust, Birmingham, UK

##### **Correspondence:** Teresa A Williams (Teresa.Williams@curtin.edu.au)

**Background**

Early identification and treatment of critical illness in the prehospital setting may improve patient outcomes. We aimed to identify prehospital factors that were predictive of critical illness requiring ICU admission, and assessed whether the Scottish Early-Warning-System (SEWS) was useful in predicting subsequent critical illness.

**Materials and methods**

After obtaining ethics approval, prehospital data of patients (≥16 years-old) transported to a public emergency department (ED) by ambulance services, between July 2012 and December 2014, were linked to the statewide ED information system using probabilistic and deterministic linkage. We excluded patients with out-of-hospital cardiac arrest and rural, interhospital, non-emergency, or air transfers. Logistic regression was used to assess whether age, presenting problem, urgency for treatment, and the first prehospital blood pressure, heart rate, respiratory rate, oxygen saturation, conscious state, temperature, and the SEWS were predictive of subsequent ICU admission.

**Results**

Of the 191,682 patients included, 2,087 (1.1%) were admitted to ICU after ED presentation. Apart from gender, all predefined predictors and the SEWS were associated with subsequent ICU admission (p < 0.001). The area under the receiver-operating-characteristic curve for the final multivariate predictive model was 0.876 (95% CI 0.865-0.886, p < 0.001); and using 1.5% predicted risk as a cut-point had a reasonable sensitivity (76%) and specificity (84%) in predicting critical illness. The mean SEWS for those requiring ICU was 5.1 compared to 1.2 for those not requiring ICU (p < 0.001).

**Conclusions**

Prehospital data appeared useful in predicting critical illness, but further refinements are needed to develop an accurate prehospital critical illness prediction model.

## A40 Intoxication and emergency response in medical emergency calls

### Thea Palsgaard Møller*, Søren Viereck, Fredrik Folke, Freddy Lippert

#### Emergency Medical Services, Copenhagen and University of Copenhagen, Copenhagen, Denmark

##### **Correspondence:** Thea Palsgaard Møller (thea.palsgaard.moeller@regionh.dk)

**Background**

Intoxication is a frequent encountered problem in emergency calls and emergency medical services (EMS). We examined the use of intoxication as category in our priority dispatch tool, analyzing underlying toxic agents, gender and age differences, emergency response provided by medical dispatchers, and temporal variation in a two year study period in the Capital Region of Denmark.

**Materials and methods**

Descriptive statistics was performed with data obtained from the database in EMS Copenhagen.

**Results**

Of 211,193 emergency calls, 12,903 (6%) were identified as intoxication related. Alcohol counted for 9,251 calls (72%), unspecified tablets for 1,733 calls (13%), narcotic drugs for 1,258 calls (10%), paracetamol for 342 calls (3%), and unspecified problems for 319 calls (2%). Highest priority response with lights and siren was provided in 17% of calls, priority 2 in 54% of calls and medical advice in 34% of calls. The number of calls was 10% in weekdays, and increased on Fridays (16%), Saturdays (24%) and Sundays (21%). Diurnal variation was identified with 38% and 42% occurrence in evening and nighttime, respectively. Adults represented the majority of calls with 34%, 32%, and 26% occurrence in the age-groups 16-30 years, 31-50 years, and 51-70 years, respectively. No gender difference was identified.

**Conclusions**

Intoxication represented 6% of all emergency calls, of which alcohol counted for 72%. Overall the intoxication contacts required an emergency ambulance in 3/4 of calls. Intoxication had the highest occurrence in weekends during evening and night time. Intoxication and especially alcohol has a major impact on utility of ambulance services.

## A41 Social inequalities in medical emergency calls

### Thea Palsgaard Møller^1^*, Annette Kjær Ersbøll^2^,Thora Majlund Kjærulff^2^, Doris Østergaard^3^, Janne Shurmann Tolstrup^2^, Jon Trærup Andersen^4^, Jerry Overton^5^, Lars S. Rasmussen^6^, Fredrik Folke^1^, Freddy Lippert^1^

#### ^1^Emergency Medical Services, Copenhagen and University of Copenhagen, Copenhagen, Denmark; ^2^National Institute of Public Health, University of Southern Denmark, Copenhagen, Denmark; ^3^Copenhagen Academy for Medical Education and Simulation, Herlev Hospital, University of Copenhagen, Copenhagen, Denmark; ^4^Laboratory of Clinical Pharmacology, Copenhagen University Hospital Rigshospitalet, Copenhagen, Denmark; ^5^International Academies of Emergency Dispatch, Salt Lake City, Utah, USA; ^6^Centre of Head and Orthopaedics, Copenhagen University Hospital Rigshospitalet, Copenhagen, Denmark

##### **Correspondence:** Thea Palsgaard Møller (thea.palsgaard.moeller@regionh.dk)

**Background**

Inequality in access to healthcare is a challenge. Medical emergency calls can be considered as access point to pre-hospital emergency care and hospital admissions. However, no data on inequality in emergency calls have been reported. Our aim was to evaluate the association between citizens’ socioeconomic position (SEP) and first-time emergency call in the Capital Region of Denmark.

**Materials and methods**

We performed an observational register-based study of 1,396,881 citizens aged 18 years or older in the Capital Region of Denmark in a two year study period (1.12.2011-30.11.2013). The Emergency Medical Dispatch Center data were linked with the Civil Registration System, Danish registers on personal labor market affiliation, education and income, and the National Patient Register. The association between SEP (exposure) and first-time emergency call (outcome) was evaluated in a logistic regression model, calculating crude and adjusted odds ratios (OR). SEP included educational level, household income and employment status.

**Results**

A total of 80,829 persons (5.79% of the study population) had an eligible first-time emergency call. Low SEP was associated with a higher probability of a first-time emergency call, with OR for the model adjusted for age, sex, marital status, comorbidity and origin as follows: Unemployed vs. employed: OR 2.46 (95% CI 2.40-2.53), lowest income quintile vs. highest income quintile: OR 1.16 (1.12-1.19), no education vs. medium or long education: OR 1.49 (1.45-1.53).

**Conclusions**

Low SEP was associated with a higher probability of first-time emergency calls indicating social inequality in emergency call patterns. Reasons for the identified inequalities need to be explored.

## A42 Quality of Danish CPR courses for laypersons

### Theo Walther Jensen^1^*, Thea Palsgaard Møller^1^, Søren Viereck^1^, Jens Roland^2,4^, Fredrik Folke^1^, Jens Flensted Lassen^3,4^, Doris Østergaard^5^, Freddy Lippert^1,4^

#### ^1^Emergency Medical Services Copenhagen, University of Copenhagen, Copenhagen, Denmark; ^2^ERC CPR-AED-group Denmark - The Danish Emergency Management Agency, Birkerød, Denmark; ^3^Department of Cardiology, Aarhus University Hospital, Aarhus, Denmark; ^4^Danish Resuscitation Council, Copenhagen, Denmark; ^5^Copenhagen Academy for Medical Education and Simulation, Herlev Hospital, University of Copenhagen, Copenhagen, Denmark

##### **Correspondence:** Theo Walther Jensen (theo.walther.jensen.01@regionh.dk)

**Background**

Bystander cardiopulmonary resuscitation (CPR) is important for survival from out-of-hospital cardiac arrest. Increase in bystander CPR is associated with increased survival. The quality of bystander CPR is likely affected by the quality of Basic Life Support (BLS) courses. The purpose of this study was to investigate the quality and adherence to international guidelines in Danish BLS courses.

**Materials and methods**

Based on previous models and elements important for survival, a course evaluation sheet was developed. It included 23 elements on a five point Likert scale incorporating theoretical, technical- and non-technical skills. Six experienced BLS instructors from various organizations where trained in the use of the evaluation sheet.

**Results**

A total of 59 course observations where conducted with the ten of the largest national BLS providers. The results of the observations showed variation between courses in most elements. All examined courses had an announced length of three to four hours. Of the observed courses 36% of had less than one hour of practical training. Most noticeable were the variation within recoil, agonal breathing, check for consciousness, telephone assisted CPR, and AED use. The preliminary results indicate an association between variation in course elements and number of participants per instructor and the length of practical training.

**Conclusions**

Variation in adherence to international guidelines, content, and quality has been documented preliminary in Danish BLS courses and can be used to improve future BLS courses.

## A43 The on-scene time in prehospital stroke care - a prospective observational study

### Tuukka Puolakka*, Sami Länkimäki, Jyrki Puolakka, Juhana Hallikainen, Kirsi Rantanen, Markku Kuisma

#### Helsinki University Hospital, Helsinki, Finland

##### **Correspondence:** Tuukka Puolakka (tuukka.puolakka@hus.fi)

**Background**

The on-scene time (OST) has been recognized as one of the most time consuming phases of prehospital stroke care, but it should not exceed 15 minutes according to the most recent American Stroke Association prehospital guidelines. We designed a training package aimed at emergency medical services (EMS) staff to cut the current OST in our system by two minutes.

**Materials and methods**

All suspected stroke patients transported to the Helsinki University Hospital before and after the completion of EMS stroke training package were recruited for this prospective observational study.

**Results**

A preliminary sample of 734 patients [49% male, mean age 66 (17) years] was recruited for the study. 429 (58%) patients were managed by the EMS before and 305 (42%) after the implementation of the training package. The ambulance dispatch was made using the stroke code in 535 (73%) cases. The EMS responded to the scene in 8 (6-10) and reached the patient in 10 (8-13) minutes after the dispatch. The OST was similar before [25 (20-31) minutes] and after [25 (19-32) minutes] the training package (p = 0.493). A physician was consulted via telephone in 412 (56%) cases. The OST duration was longer in these calls [27 (21-34) minutes] when compared to situations where no consultation was made [23 (18-28) minutes] (p < 0.0001).

**Conclusions**

The implementation of a training package designed to decrease the OST had no effect to the median duration of the on-scene stay. Consulting a physician was related to increased OST duration which necessitates further analysis.

